# Tailorable porous collagen hydrogels as a physiologically relevant platform for extrachromosomal DNA-associated colorectal cancer research

**DOI:** 10.7150/thno.128574

**Published:** 2026-03-09

**Authors:** Seoyul Jo, Jiwon Shon, Seohyeon An, Yoonjoo Nam, Dongwon Choi, Seungmi Lee, Hoigi Seo, Se Young Chun, Hoon Kim, GeunHyung Kim

**Affiliations:** 1 Department of Precision Medicine, Sungkyunkwan University School of Medicine (SKKU-SOM), Suwon 16419, South Korea.; 2 Department of Biohealth Regulatory Science, School of Pharmacy, Sungkyunkwan University, Suwon, South Korea.; 3 Department of Biopharmaceutical Convergence, School of Pharmacy, Sungkyunkwan University, Suwon, South Korea.; 4 Department of Pharmacy, School of Pharmacy, Sungkyunkwan University, Suwon-si, South Korea.; 5 Department of Electrical and Computer Engineering, Seoul National University, Seoul, South Korea.; 6 INMC &amp; IPAI, Seoul National University, Seoul, Korea.; 7 Institute of Quantum Biophysics, Department of Biophysics, Sungkyunkwan University, Suwon, Gyeonggi-do 16419, South Korea.; 8 Biomedical Institute for Convergence at SKKU (BICS), Sungkyunkwan University, Suwon 16419, South Korea.

**Keywords:** tumor microenvironment (TME), extrachromosomal DNA (ecDNA), collagen hydrogel, porous structure, 3D *in vitro* model

## Abstract

A novel 3D porous hydrogel model, mimicking the tumor microenvironment (TME), was developed as a physiologically relevant platform to investigate the role of extrachromosomal DNA (ecDNA) in colorectal cancer. We fabricated a tailorable collagen-based hydrogel that overcomes the limitation of 2D cultures by enabling crucial cell-cell and cell-matrix interactions.

**Methods:**

First, we validated whether the selected COLO320 cell lines were suitable for investigation of ecDNA in 3D tumor model and confirmed that ecDNA structures were stably maintained under 3D culture conditions by whole genome sequencing (WGS). Additionally, to provide an appropriate environment for colorectal cancer cells, we fabricated collagen-based porous hydrogels using a whipping process that requires no surfactants or sacrificial materials. During this process, we optimized the bioink formulation to achieve extracellular matrix (ECM) stiffness favorable for colorectal cancer cell proliferation, aggregation, stem-like behavior, and epithelial-mesenchymal transition (EMT)-related gene expression.

**Results:**

By optimizing the porous structure for enhanced nutrient diffusion and cell infiltration, we successfully maintained ecDNA structures in COLO320 cells. Our optimized porous platform significantly enhanced cellular proliferation, aggregation, and metabolic activity compared to conventional bulk model. We also observed elevated expressions of key oncogenes like MYC and activation of mechanotransduction pathways associated with aggressive tumor phenotypes.

**Conclusion:**

This reproducible and effective model accurately reflects ecDNA-driven biological behaviors, making it ideal for long-term ecDNA research and future TME-related studies.

## Introduction

The tumor microenvironment (TME) refers to the surrounding niche of tumors, consisting of extracellular matrix (ECM) components, stromal and immune cells, and soluble factors that collectively regulate cancer 1, 2. Because of its central role in tumor growth, invasion, and therapeutic response, the development of physiologically relevant TME models has been a major focus in cancer research. Traditionally, animal models such as mice and zebrafish provide valuable insights into tumor initiation, angiogenesis, and immune interactions, but their interspecies differences and limited experimental controllability reduce reproducibility 3-5.

To address these limitations, engineered *in vitro* TME models have been developed for drug screening, mechanistic studies, and immunotherapy evaluation 6-18. Microfluidic organ-on-chip systems enable precise control of gradients and fluidic environments, but their conventional two-dimensional (2D) structures fail to capture native three-dimensional (3D) architecture and cell-ECM interactions, limiting physiological relevance. More recently, hydrogel-based 3D tissue models have emerged as promising alternatives, providing tailorable biochemical and mechanical cues that better recapitulate *in vivo* tissue function (Table S1). For example, 3D bioprinted colorectal cancer models for chemotherapeutic screening, hyaluronic acid/gelatin organoid co-cultures for studying colorectal cancer cell-ECM interactions, and alginate cryogels for immunotherapy evaluation have all been developed 15-17.

In the fabrication of hydrogel-based 3D TME models, the porous structure of the matrix is a critical physical feature 19, 20. Generally, interconnected pore networks in the cell-culture structures enhance nutrient diffusion, immune cell infiltration, and tumor-like cellular behavior 21-25. In particular, pore size, defined as the bubble diameter in this study, plays a key role, as it directly affects cell migration, proliferation, and vascularization. Previous studies suggest that interconnected pores of ~150-400 μm can promote vascular infiltration and nutrient exchange 20. However, most conventional cryogel-based porous TMEs rely on post-seeding methods, often leading to uneven cell distribution and limited reproducibility 26-28. This highlights the need for cell-laden porous models that simultaneously provide structural integrity and tailorable mechanical cues optimized for tumor progression.

**Figure 1:**
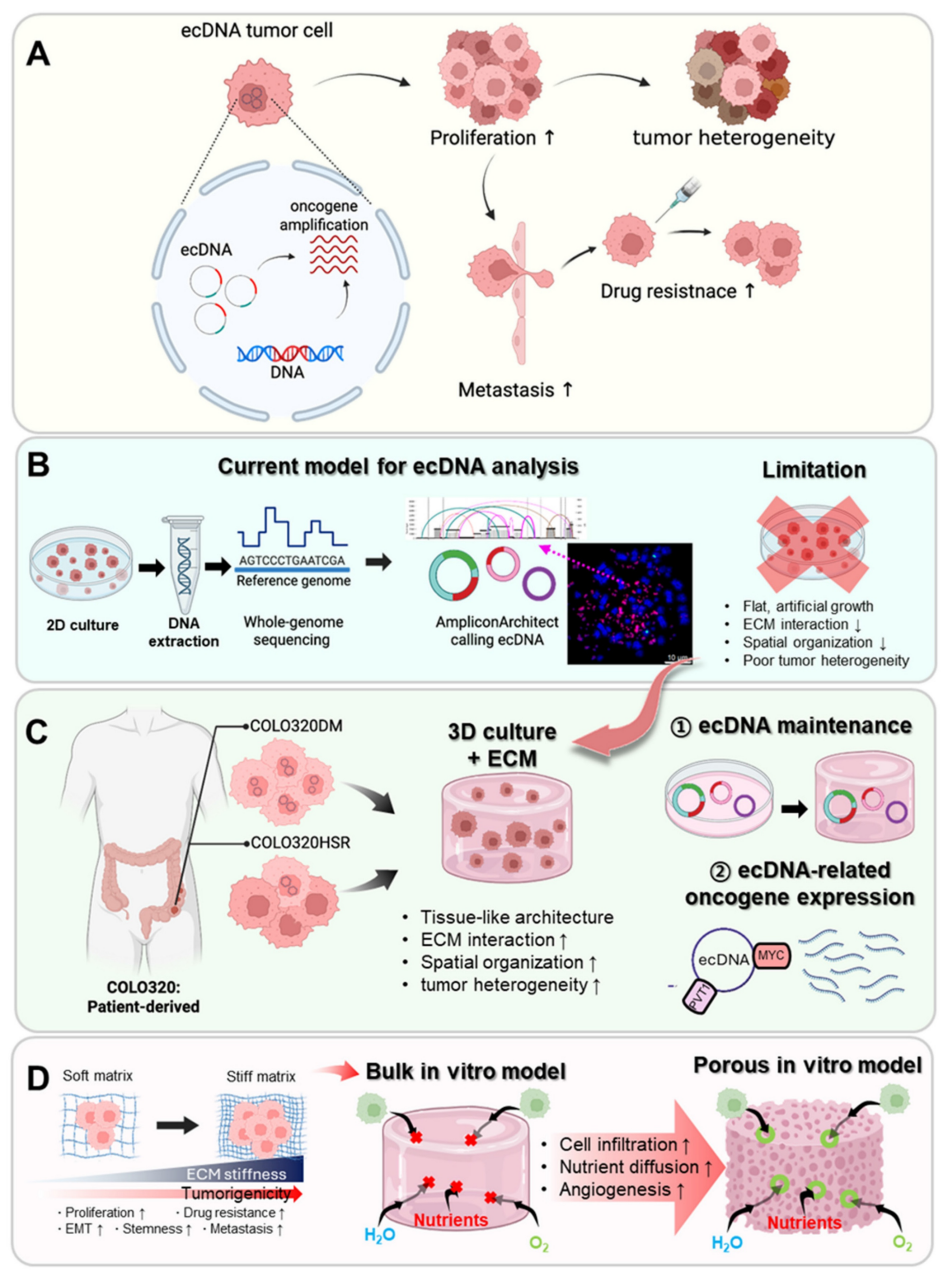
** Schematic of experimental design using porous collagen bioinks for colorectal cancer modeling.** (**A**) Concept of ecDNA arising from focal rearrangements and carrying amplified oncogenes (e.g., *MYC*) in tumor cells. EcDNA enabling rapid copy-number change and uneven mitotic segregation, driving proliferative advantage, intratumoral heterogeneity, therapy resistance, and metastatic potential (**B**) EcDNA detection workflow showing genomic DNA extraction from 2D cultured cancer cells and its limitation. Whole-genome sequencing, read alignment to the reference genome (GRCh37), and amplicon reconstruction with AmpliconArchitect. AmpliconClassifier-based classification of ecDNA versus ChrAmp based on cyclic topology, and representative FISH images using a MYC probe showing multiple extrachromosomal foci, consistent with MYC amplification on ecDNA. (**C**) Model system using Patient-derived COLO320DM (double-minutes; ecDNA-positive) and COLO320HSR (chromosomal HSR) colorectal cancer lines, cultured in 3D systems with ECM. Tissue-like architecture, stronger cell-ECM interactions, improved spatial organization, and greater tumor heterogeneity in 3D+ECM compared with 2D (**D**) Development of a porous collagen bioink-based tumor model system that reflects tumor ECM characteristics while overcoming the limitations of bulk hydrogels.

Recent genomic studies have revealed that extrachromosomal DNA (ecDNA) plays a pivotal role in tumor proliferation, recurrence, and metastasis 29-33. EcDNA, often observed as double minutes (DMs), are acentromeric and atelomeric circular DNA elements that serve as carry amplified oncogenes, thereby driving heterogeneity and therapy resistance (Figure 1A) 33, 34. They are prevalent across multiple solid primary and metastatic tumors—including glioblastoma (65-76% of cases harboring ecDNA), breast cancer (37-47%), lung cancer (21-32%), and colorectal cancer (CRC; 3-18%)—and are consistently associated with poor patient outcomes 18. Traditional ecDNA studies have relied on 2D cultures, organoids, and mouse models 35-38; however, these systems inherently fail to reproduce complex ECM interactions and the architecture of human tumors (Figure 1B) 35. Consequently, 3D hydrogel-based models can offer a more reproducible and physiologically relevant platform for investigating ecDNA functions within the TME (Figure 1C) 39, 40.

In this study, we focus on the COLO320 colorectal cancer cell line, which originates from a single patient but contains two subclones: COLO320-DM, predominantly characterized by ecDNA amplification, and COLO320-HSR, by chromosomal amplification (Figure 1C). These isogenic cell lines provide a unique opportunity to directly compare the biological consequences of ecDNA versus chromosomal amplification in an identical genetic background 41, 42. As colorectal cancer frequently exhibits ecDNA-driven oncogene amplification, this model is well suited for studying ecDNA-associated tumor behaviors.

Tumor progression in colorectal cancer is also strongly influenced by ECM stiffness, which regulates migration, invasion, and epithelial-mesenchymal transition through mechanotransduction pathways 43-45. Cancer stem cells, for example, show enhanced growth and stemness marker expression on substrates of ~25 kPa stiffness 46, 47. Previous attempts to mimic these mechanical properties have mainly used stiff 2D substrates or compressed hydrogels 46-51, but dense or non-porous bulk hydrogels, particularly those used to achieve high stiffness, can suffer from poor oxygen and nutrient diffusion, thereby compromising physiological fidelity compared to porous structure 52, 53.

Here, we propose a porous collagen-based hydrogel TME model that integrates interconnected pore networks for improved diffusion with tailorable stiffness to better replicate *in vivo* tumor mechanics (Figure 1D). By engineering collagen hydrogels with identical pore architectures but varying stiffness, we sought to identify the conditions most conducive to ecDNA-driven cancer behaviors. Using COLO320-DM and COLO320-HSR cells, we demonstrate that this platform supports stable ecDNA maintenance, promotes aggregate formation, and enhances oncogene transcription compared with conventional 2D or bulk hydrogel systems.

## Experimental Methods

### Cell cultivation and bioink preparation

COLO320-DM and COLO320-HSR colorectal cancer cell lines (Korean Cell Line Bank, South Korea) were cultured in RPMI1640 medium (Welgene Inc., South Korea) supplemented with 10% fetal bovine serum (FBS; Biowest, Riverside, MO, USA) and 1% penicillin-streptomycin (PS; Thermo Fisher Scientific, Waltham, MA, USA). Cells were cultured in a humidified incubator at 37 °C with 5% CO₂, and the culture medium was replaced every 2-3 days. For 3D encapsulation, harvested cells were mixed with collagen-based bioinks at a final density of 1 × 10⁷ cells/mL. Briefly, 1 × 10⁷ cells were first resuspended in 20 μL of culture medium to prepare a cell suspension. The cell suspension was then gently mixed with the bioink using a syringe and a 3-way stopcock by passing the mixture back and forth more than 20 times to ensure homogeneous cell distribution.

Bulk-type collagen bioinks were formulated using atelocollagen type I derived from porcine skin (MSBio, Seongnam, South Korea), initially supplied as an acidic solution (pH 4). The collagen was lyophilized, redissolved in triple-distilled water (3DW), and neutralized using 10× concentrated culture medium (pH ~7) to obtain final concentrations of 2 w/v% and 3.5 w/v%, as previously described 54. To fabricate porous collagen foam bioinks, collagen hydrogel (5 w/v%) was first diluted with the 1 mM genipin solution to reach a final concentration of 3.5 w/v%. The resulting mixture was aerated using an automatic stirring system equipped with a custom-designed 3D-printed whipper (fitting a 50 mL conical tube) operated at 2000 rpm for up to 30 min, until the volume reached saturation through uniform air incorporation 55. The cell-laden collagen foam bioink was injected into cylindrical molds and incubated in the culture media containing genipin (1 mM) for 1 h at 37 °C for additional crosslinking.

### Characterization of collagen foam bioinks

The porous microstructure of the collagen foams after whipping was visualized using an optical microscope (BX FM-32; Olympus, Tokyo, Japan). Bubble diameters were quantified from the acquired images using ImageJ software (NIH, Bethesda, MD, USA). To determine the air volume fraction, both foam and bulk collagen hydrogels of identical concentration were molded into cylindrical shapes (diameter: 1 cm, height: 8 mm), and the weight difference between the two was measured. This difference was converted into volume and used to estimate the incorporated air content.

The rheological characteristics of collagen foams were evaluated before and after crosslinking using a rotational rheometer (Bohlin Gemini HR Nano, Malvern Instruments) equipped with a 40 mm diameter cone-and-plate system (parallel, 1 mm gap). A frequency sweep (1 - 100 rad/s) were carried out at 25 °C under a fixed strain (1%), within the linear viscoelastic regime. A temperature sweep was performed from 10 °C to 45 °C at a rate of 2 °C/min with 1% strain at 1 Hz.

Mechanical characterization of the constructs was performed under wet conditions using a universal testing machine (SurTA; Chemilab, South Korea) in compressive mode with a loading rate of 0.1 mm/s. Stress-strain curves were obtained, and the apparent modulus was calculated from the linear region of the curve. The inherent modulus was further estimated using the Gibson-Ashby model 56.

### *In vitro* cellular activity evaluations

Cell viability within the collagen-based porous scaffolds was evaluated after 1 day of culture using a live/dead viability assay. Constructs were incubated in PBS containing 0.15 μM calcein-AM and 2 μM ethidium homodimer-1 (Thermo Fisher Scientific, USA) for 30 min at 37 °C. Live and dead cells were visualized as green and red fluorescence signals, respectively, using a confocal laser scanning microscope (LSM 700; Carl Zeiss, Germany). Cell viability was quantified by calculating the ratio of live cells to total cells using ImageJ software (NIH, USA).

Cell proliferation was assessed on days 1, 3, and 7 using the Cell Counting Kit-8 (CCK-8; Dojindo, Japan). After washing the constructs with PBS, samples were incubated in a 10-fold diluted CCK-8 solution in DMEM at 37 °C for 2 h under 5% CO₂. The resulting colorimetric change was quantified by transferring 200 μL of the supernatant to a 96-well plate, and absorbance was measured at 450 nm using a microplate spectrophotometer (Agilent Technologies, Santa Clara, CA, USA).

Cell apoptosis was assessed on days 1, 3, and 7 using the Caspase-3 Activity Assay Kits (ThermoFisher, USA). Each sample was lysed in lysis buffer for 30 min on ice. Then, the fluorogenic caspase substrate was added to the lysates, and fluorescence was measured at 342 nm excitation and 441 nm emission using HTS Multi-mode microplate reader (Agilent, USA).

To evaluate cell aggregation behavior, constructs were fixed and stained with DAPI (Invitrogen, USA) after 3, 7, 14, 21, and 28 days of culture. Aggregation size and area were measured using confocal microscopy (LSM 700; Carl Zeiss) and analyzed with ImageJ software.

### Real-time polymerase chain reaction (RT-PCR)

Quantitative reverse transcription PCR (qRT-PCR) was conducted to assess the expression of ecDNA-associated genes (*PVT1, MYC, POU5F1B*, and *FAM84B*), cancer stem cell markers (*CD133* and *CD44*), proliferation marker (Ki67), and EMT-related markers (Vimentin and N-cadherin). Total RNA was isolated from the cell-laden constructs using Easy-BLUE™ reagent (Intron Biotechnology, South Korea) according to the manufacturer's protocol. The extracted RNA was reverse transcribed into complementary DNA (cDNA) using the ReverTra Ace® qPCR RT Master Mix (Toyobo, Osaka, Japan). Quantitative PCR was performed using Thunderbird® SYBR® qPCR Mix (Toyobo) on a StepOnePlus™ Real-Time PCR System (Applied Biosystems, Waltham, MA, USA). Relative gene expression levels were determined using the 2^-ΔΔCT method and normalized to the expression of the housekeeping gene GAPDH. Gene-specific primer sequences (Bionics, Seoul, South Korea) are provided in Table S2. Following 30 amplification cycles, PCR products were stained with Loading STAR dye (Dyne Bio, South Korea) and separated by electrophoresis on a 2% agarose gel. All uncropped gel electrophoresis images are presented in Figure S8-S13.

### Immunofluorescence staining

Immunofluorescence staining was conducted to evaluate the expression of oncogenic and proliferative markers in cancer cells. Specimens were washed twice with PBS and fixed with 3.7% formaldehyde in PBS at 37 °C for 30 min. Fixed samples were permeabilized and blocked by sequential incubation with 2% Triton X-100 (Cytiva Hyclone Laboratories, USA) and 2% bovine serum albumin (BSA; Sigma-Aldrich, USA) at 37 °C for 2 h each. Following blocking, samples were incubated overnight at 4 °C with primary antibodies against MYC and Ki67 (1:200 dilution in PBS; Invitrogen, USA). After washing, Alexa Fluor 488- and 594-conjugated secondary antibodies (1:50 dilution in PBS; Invitrogen) were applied for 1 h at room temperature. Cell nuclei were counterstained with DAPI. Fluorescence images were acquired using a confocal laser scanning microscope (LSM 700; Carl Zeiss, Germany).

### FISH imaging and ecDNA quantification

Metaphase FISH was performed to assess extrachromosomal *MYC* signals in 2D samples. Cells were arrested in metaphase with KaryoMAX Colcemid (Gibco, 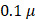
m/mL, 4h), treated hypotonic treatment with 0.075 M KCL (15 min, 37 °C), and fixed in Carnoy's fixative (methanol:glacial acetic acid, 3:1) with three changes. Single-cell suspensions were prepared in fixative and dropped onto glass slides. Fixed samples were equilibrated in 2x SSC and sequentially dehydrated in 70%, 85%, and 100% ethanol (2 min each). *MYC* and control probes (Empire Genomics, MYC-20-OR and CHR08-20-GR) were denatured at 75 °C for 3 min and applied to the samples. After overnight hybridization, slides were washed with 0.4x SSC and 2x SSC/0.05% Tween-20 (2 min each) and counterstained with DAPI (Vectashied, H-200). FISH images were acquired using a Leica STELLARIS 5 microscope with a 63x oil immersion objective.

Quantification was performed with a custom RGB segmentation script on three-channel TIFFS (red = *MYC* FISH, green: chr8 centromere probe, blue: DAPI). Red signals overlapping nuclei or chr8 masks were removed; per-cell outputs included the number of extrachromosomal MYC foci and chr8 features (object counts, area 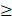
5px; total area; mean intensity). Counts were normalized to two chr8 objects per cell, with all parameters hold constant across samples.

### DNA extraction and whole-genome sequencing (WGS)

Genomic DNA was collected from COLO320-DM and COLO320-HSR under two conditions, conventional 2D monolayer and a 3D collagen scaffold-based model. For 2D samples, Illumina paired-end libraries were prepared with the TruSeq Nano DNA Sample Prep Kit and sequenced to 90-95 Gbp per sample (DM_2D, 90.23 Gbp;HSR_2D, 95.07 Gbp). For 3D samples, the libraries used in quantitative analyses were prepared with TruSeq Nano and yielded the following totals: DM_3D-1, 97.50 Gbp; DM_3D-2, 96.15 Gbp; HSR_3D-2, 91.17 Gbp;HSR_3D-2, 103.28 Gbp. A separate DM/HSR pair not used for quantitative analyses was prepared with the QIAseq FX DNA Library Kit, yielding 30.08 Gbp and 35.57 Gbp;this pair was retained for qualitative concordance checks only.

### Read processing and quality control

Reads were aligned to GRCh37 with BWA-MEM, coordinate-sorted, and duplicates marked with Picard MarkDuplicates. Base quality score recalibration (BQSR) was performed using GATK 4.1.8.1. Preliminary coverage summaries were generated with GATK DepthOfCoverage. Global post-alignment QC including coverage, purity, ploidy estimation and allele-specific copy number (COBALT/PURPLE) followed nf-core/oncoanalyser v1.0.0 defaults. Sample identity was verified using Picard CrosscheckFingerprints and NGSCheckMate v1.0.1, only samples passing both checks were retained for downstream analyses (Figure S1C-E).

### Amplicon reconstruction

We used AmpliconArchitect (AA; AmpliconSuite-pipeline v0.1344.2) on GRCh37 with default parameters except for seed-detection depth. Candidate seed regions were defined by the pipeline (CNVkit 57) using copy number 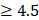
and length 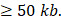
To harmonize depth across samples, BAMs were temporarily downsampled to 20x for seed detection only. The final amplicon reconstruction used the full, non-downsampled BAM. Post-reconstruction, AmpliconClassifier (v0.4.12) assigned focal amplifications to circular (ecDNA) or linear (ChrAmp) classes and refined gene coordinates, only high-confidence calls were retained (https://github.com/AmpliconSuite/AmpliconSuite-pipeline). Complete Reconstruction of Amplifications with Long reads (CoRAL) was used to visualize the resulting amplicon structures (Figure S4A-B).

### Amplicon complexity and similarity

Amplicon complexity was summarized using the entropy-based complexity score implemented in AmpliconClassifier, computed from AA's cycles output (copy-number-aware breakpoint graph). Briefly, the score aggregates the length-weighted distribution of copy-number flow across genome paths and includes a residual component for copy number not explained by dominant path, higher value indicates more structurally complex amplicons. For ross-sample comparisons, we used amplicon_similarity.py (AmpliconClassifier v0.4.12). For overlapping amplicon pairs, the tool computes a similarity score that jointly considers shared genomic content and shared breakpoints and evaluates significance against a background of unrelated overlapping pairs; 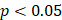
was considered evidence of as shared event.

### Statistical analysis

All quantitative data are presented as mean ± standard deviation (SD). Statistical analyses were performed using SPSS software (SPSS Inc., USA) and Python/SciPy. For comparisons between two groups, Student's t-test was employed. The distributions of aggregation sizes for all cell aggregates in each group were statistically analyzed using the Kolmogorov-Smirnov test. For comparisons among more than three groups, one-way analysis of variance (ANOVA) followed by Tukey's honestly significant difference (HSD) post hoc test was conducted. To assess monotonic increases across the ordered 3D series, we applied a rank-based trend screen using Spearman's rank correlation (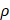). Statistical significance was defined as p* < 0.05, p** < 0.005, and p*** < 0.0005.

## Results

### Validation of COLO320 cell lines containing ecDNA

To utilize COLO320 cells in this study, we confirmed the ecDNA and chromosomal amplification status of the isogenic COLO320-DM (DM_2D) and COLO320-HSR (HSR_2D) cell lines under conventional 2D culture conditions using whole genome sequencing-based analyses and imaging. This baseline comparison confirmed that their distinct amplification patterns were preserved in 2D, thereby providing a reference for evaluating whether these features are maintained in 3D culture.

**Figure 2:**
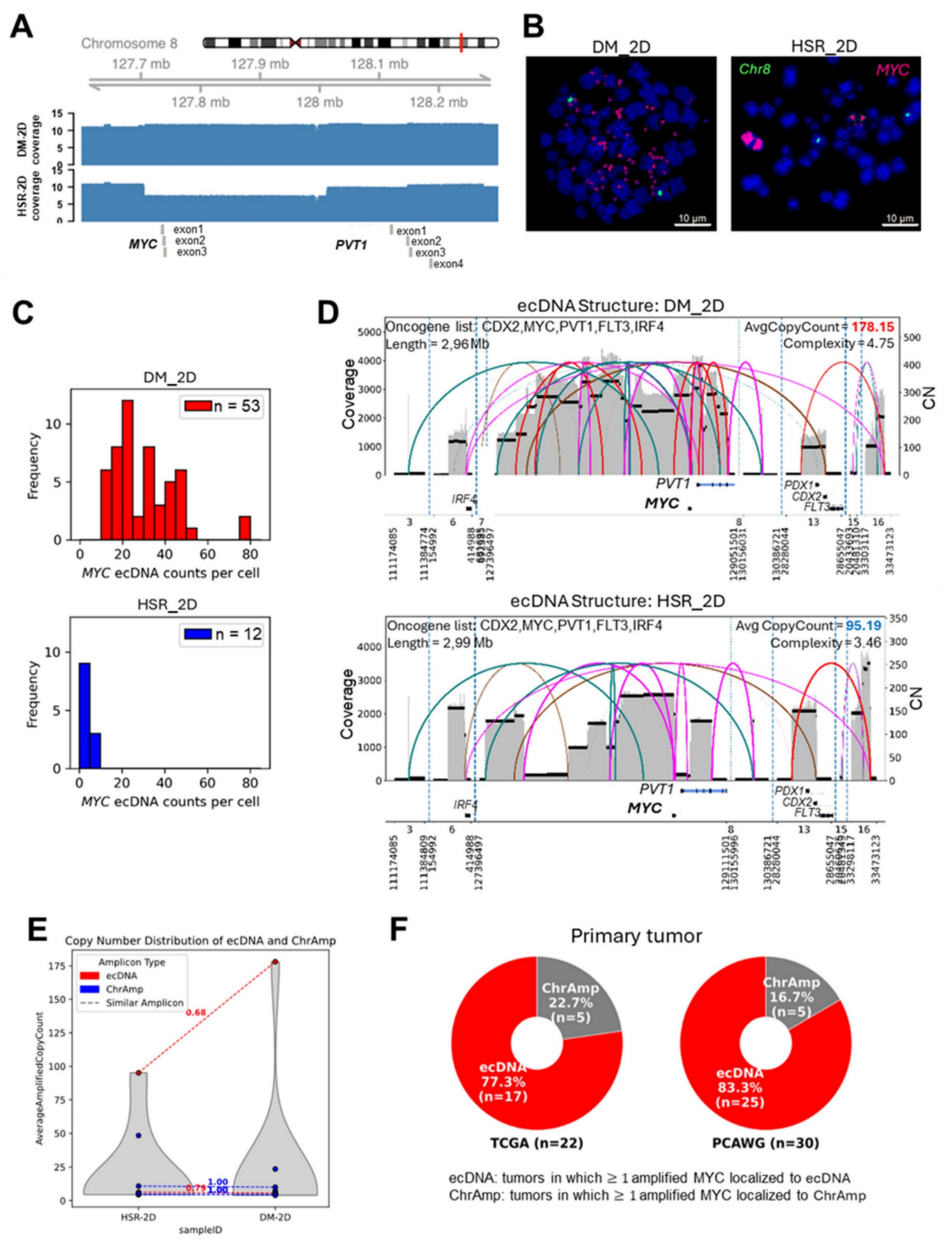
** Characterization of *MYC* amplicon structures in 2D-cultured COLO320 cells and patient tumor.** (**A**) Log_2_ scaled copy-number profile across chr8 (127.7-128.2 Mb) in 2D-cultures. Discrete peaks align with *MYC* exons 1-3 and *PVT1* exons 1-4; COLO320-DM shows constantly higher coverage than COLO320-HSR. (**B**) Representative metaphase FISH images using a *MYC* (red), chromosome 8 centromere (green), DAPI (blue), and scale bar, 10 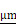. COLO320-DM displays numerous extrachromosomal *MYC* foci, whereas COLO320-HSR shows predominantly intrachromosomal HSR-like bands. (**C**) Distribution of per-cell *MYC* ecDNA in 2D (DM_2D, n = 53 HSR-2D, n = 12). DM_2D exhibits a broad, high-count range, whereas HSR_2D rarely shows more than a few copies. Quantified by a uniform segmentation script. (**D**) AmpliconArchitect reconstructions of *MYC*-containing ecDNA in both samples. Structural similarity between cell lines is moderate (SimilarityScore = 0.68). Panel annotations report Oncogene list/Length (top left) and AverageCopyCount/Complexity (top right) (**E**) Average amplified copy-count distributions per amplicon (ecDNA vs ChrAmp). Shared non-*MYC* amplicons cluster at low copy number, whereas the *MYC*-containing ecDNA dominated the copy-number burden in both cells. Dashed guides describe paired amplicon between DM_2D and HSR_2D and the values show similarity score. (**F**) Proportion of *MYC*-amplified primary tumors in TCGA (n = 22) and PCAWG (n = 30), classified as *MYC*-ecDNA (red) and *MYC*-ChrAmp (gray). Denominators include only tumors with confirmed *MYC* amplification and high confidence ecDNA versus chromosomal call per cohort.

Copy-number profiles depicted a focal amplification spanning *MYC* proto-oncogene (*MYC,* exons 1-3) and *Plasmacytoma variant translocation 1* (*PVT1,* exon1-4*)*, a long non-coding RNA (lncRNA) frequently co-amplified with *MYC,* in DM_2D cells, showing consistently elevated copy-number segment across the *MYC-PVT1* locus (Figure 2A), which is characteristic of extrachromosomal DNA. Fluorescence In Situ Hybridization (FISH) using *MYC* probes and chromosome 8 centromere probes corroborated the copy-number profiles. In DM_2D cell line, numerous bright *MYC* signals appeared as double-minute-like foci spatially separated from chromosome 8 centromeric signals (green), indicating extrachromosomal localization. By contrast, HSR_2D cell line exhibited strong intrachromosomal *MYC* signals typical of HSRs, together with occasional small extrachromosomal spots (Figure 2B). Automated image quantification across 53 DM_2D and 12 HSR_2D metaphase showed per-cell *MYC* ecDNA counts ranging from 15 to 80 in DM_2D, while HSR_2D rarely exceeded 8 (Figure 2C). Together, the copy-number profiles and *MYC*-ecDNA quantifications indicate a broader and higher distribution of *MYC*-harboring ecDNA in DM relative to HSR under 2D culture conditions.

AmpliconArchitect, a tool for reconstructing ecDNA amplicon structures using whole genome sequencing (WGS), detected three ecDNA amplicons in each cell line, including two shared and one private amplicon per cell line (Figure 2D) 58. The most highly amplified ecDNA in both cell lines contained *MYC* (average copy number: DM_2D = 178.15, HSR_2D = 95.19) with moderate structural similarity (similarity score = 0.68, a metric quantifying overlap of genomic segments and breakpoint configurations; see experimental section) 59. Fragments derived from multiple chromosomes (chr 3, chr 6, chr 8, chr 12, chr 15, and chr 16) in both, whereas the DM_2D-specific ecDNA additionally incorporates a chromosome 7 segment. Oncogene annotations confirmed overlapping cargos, including *MYC, PVT1, IRF4* (interferon regulatory factor* 4*, transcription factor in lymphoid malignancies)*, CDX2* (caudal type homeobox 2, intestinal lineage marker), *PDX1* (pancreatic and duodenal homeobox 1, pancreatic transcription factor*)* and *FLT3* (fms-like tyrosine kinase 3, hematopoietic receptor tyrosine kinase*)*. DM_2D-specific ecDNA included oncogenes such as *CYLD* (cylindromatosis, NF-kB pathway regulator*)* but carried a low average copy-number, while HSR_2D-specific ecDNA appeared structurally simple and lacked canonical oncogenes. Both private ecDNAs showed relatively lower average copy count than *MYC*-containing ecDNA. Shared non-*MYC* ecDNA and chromosomal amplicons (ChrAmp) showed lower copy-number levels, whereas the *MYC*-containing ecDNA accounted for the dominant amplification burden in both cells (Figure 2E). DNA gene-level copy-number profiling within the *MYC* ecDNA region showed significantly higher levels of *MYC*-associated genes such as *PVT1, POU5F1B (*POU class 5 transcription factor 1B, pseudogene transcription activator*)*, *FAM84B* (family with sequence similarity 84, member B, cancer migration and invasion*)* in DM_2D (Figure S1A).

Expanding our analysis to large patient cancer cohorts, we quantified *MYC*-amplified tumors using publicly available data from PCAWG (Pan-Cancer Analysis of Whole Genomes) (n = 2,645 patients) and TCGA (The Cancer Genome Atlas) (n = 975 patients), covering 22 primary cancer types including glioblastoma, breast, and colorectal cancer 34. Among *MYC*-amplified tumors, *MYC* occurs on ecDNA: 83.3% in PCAWG and 77.3% in TCGA (Figure 2F). *PVT1*, a lncRNA known to enhance *MYC* expression, frequently co-localizes with *MYC* on the same ecDNA amplicon in 76.7% and 73.9% of cases, respectively (Figure S1B). Both cohorts were analyzed using previously published processed data 34.

Through the analysis of the 2D culture conditions, DM_2D exhibited a substantially higher burden and broader distribution of *MYC*-amplified ecDNA than HSR_2D. AmpliconArchitect-based structural reconstruction and FISH-based ecDNA quantification consistently identified *MYC* as the dominant ecDNA driver in both cell lines.

### Characterization of ecDNA structures in a collagen-based 3D culture model

While extensive research has focused on ecDNA in 2D culture systems, there is still a lack of information regarding its behavior in physiologically relevant 3D environments such as hydrogels. To address this gap, we cultured ecDNA-containing colorectal cancer cells within a 3D collagen-based hydrogel system prior to the fabrication of a tumor model and evaluated the differences in cellular behavior and ecDNA structural maintenance between 2D and 3D culture conditions.

**Figure 3:**
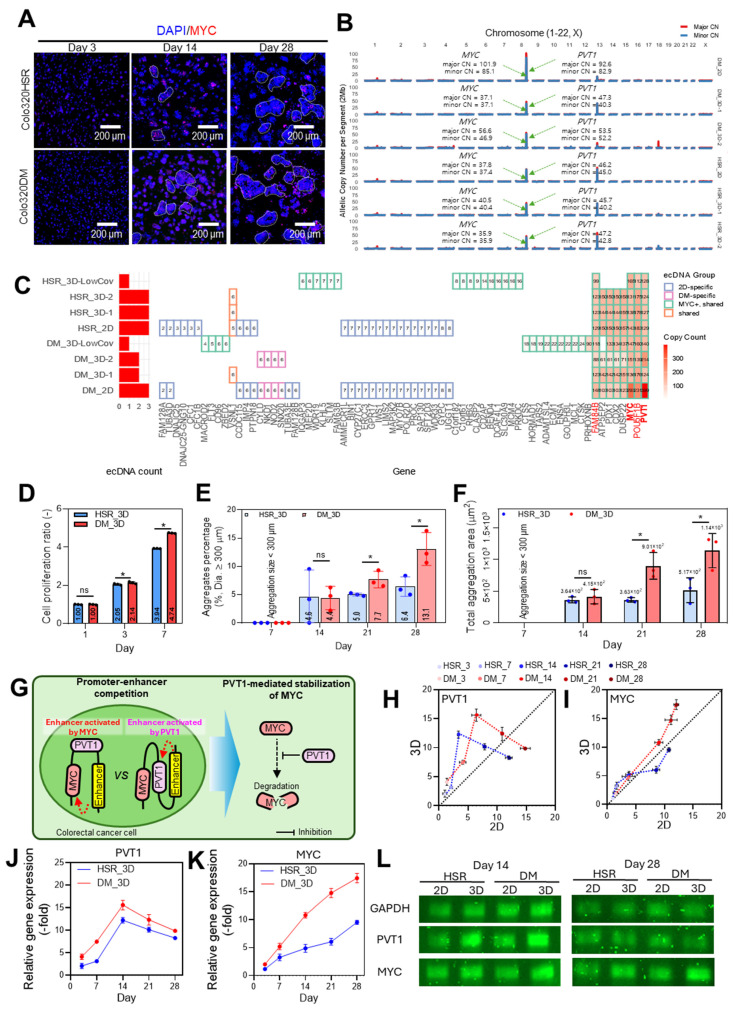
** 3D collagen hydrogel culture enhances aggregation and reveals dynamic regulation of ecDNA-associated PVT1 and MYC expression in COLO320 cells.** (**A**) Immunofluorescence staining of MYC (red) and nuclei (DAPI, blue) in COLO320-DM and COLO320-HSR cells cultured in 3D collagen hydrogels for 28 days. (**B**) Genome-wised copy number profiles (major allelic copy-number: red, minor: blue) across all chromosomes in 2D. Green arrows mark the *MYC* and *PVT1* locus on chr8, showing a focal high-level amplification while other chromosomes remain near baseline. (**C**) Oncogenes detected on ecDNA are shown for each sample. The left bar plot displays the number of ecDNA amplicons. In the heatmap, genes are grouped by ecDNA group: 2D-specific (blue), DM-specific (pink), MYC⁺ shared ecDNA (green), and shared ecDNA across multiple conditions (orange). Red fill intensity within boxes represents gene-level copy number, highlighting focal amplifications such as *MYC, PVT1, POU5F1B* and *FAM48B*. (**D**) Cell proliferation measured by CCK-8 assay on days 1, 3, and 7 (n = 3). (**E**) Aggregate percentage based on the number of aggregates with diameters larger than 300 μm in 3D cultures at days 7, 14, 21, and 28. (**F**) Total aggregation area per unit area for aggregates larger than 300 μm in diameter at days 7, 14, 21, and 28 (n = 3). (**G**) Schematic illustrating the dual regulatory role of *PVT1* in *MYC* expression: while *PVT1* and *MYC* form a positive feedback loop, inhibition of the *PVT1* promoter can paradoxically increase *MYC* expression through enhancer competition. Relative mRNA expression of (**H**) *PVT1* and (**I**) *MYC* in COLO320-DM and HSR cells cultured in 2D and 3D for 28 days (n = 3). Temporal expression patterns of (**J**) *PVT1* and (**K**) *MYC* in 3D culture at days 3, 7, 14, 21, and 28. (**L**) Gel electrophoresis showing expression levels of *PVT1* and *MYC* at days 14 and 28 in 2D and 3D culture. All values are represented as mean ± standard deviation.

We fabricated 3D collagen-based tumor constructs using collagen hydrogel embedded with either COLO320-HSR (HSR_3D) or COLO320-DM (DM_3D) cells at a cell density of 1 × 10^7^ cells/mL (Figure S2). Reproducibility is a critical evaluation criterion in the development of in vitro tumor models. To ensure reproducibility, it is essential that the model structure is stably maintained. In this study, porcine skin-derived type I collagen was used, and a collagen concentration of 20 mg/mL (2 w/v%) was selected as the minimum level required to maintain structural and mechanical stability after fibrillization (Figure S2A-B). As shown in Figure 3A, cellular aggregation within both cell-laden collagen bioinks was observed on days 3, 14, and 28 using DAPI/MYC immunofluorescence staining.

To characterize the ecDNA structures in both HSR_3D- and DM_3D-laden hydrogels, WGS was performed on three biological replicates for each cell line. All biological replicates were confirmed to originate from the same cell line using fingerprint analysis (Figure S3A-B) 60, 61. This validation ensured that replicate-level comparisons reflected true biological validation rather than sample misidentification.

One replicate from each group (DM_3D and HSR_3D) had low sequencing depth (<5×), indicating insufficient read depth for reliable quantitative comparisons. These two low-depth replicates were therefore excluded from quantitative analyses but retained for qualitative assessment to evaluate overall ecDNA structure preservation (Figure S3C). Genome-wide allelic copy-number profiles across all chromosomes showed pronounced focal amplification at the *MYC*-*PVT1* locus on chromosome 8 in both 2D and 3D cultures, whereas remaining genomic regions remained near baseline (Figure 3B). This shared focal peak corresponded to the known ecDNA locus, indicating that *MYC*-associated ecDNA is consistently maintained under both culture conditions (Figure 3C). Across 3D replicates, *MYC*-harboring ecDNA structures were consistently detected with a high structural similarity score ranging from 0.73 to 0.95, including low coverage samples. When grouped according to this metric, the 3D amplicons clustered into distinct ecDNA groups, within which *MYC*^+^ ecDNAs consistently retained their core configurations and associated gene contents including *MYC, PVT1, FOU5F1B, FAM84B*, etc. The results confirm that *MYC*-associated ecDNA structures were stably maintained across independent 3D replicates under even low sequencing depth.

Additionally, to further investigate potential ecDNA-driven phenotypic divergence among 3D cultures, we analyzed aggregation patterns in both DM_3D and HSR_3D groups (Figure S3). The formation of cell aggregates within tumors increases the probability of microvessel induction through interactions with endothelial cells and facilitates their extravasation into surrounding tissues, both of which are critical steps in the metastasis 62. Therefore, we observed that the aggregation size progressively increased from HSR_3D-1 and HSR_3D-2 to DM_3D-1 and DM_3D-2, suggesting enhanced proliferative behavior in DM cultures (Figure S3D-F). Copy-number profiling identified three 8q24 lncRNAs-*PCAT1, PRNCR1*, and *CASC19*- whose copy-number gains strongly correlated with this aggregation pattern (Spearman's 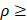
0.9, p < 0.05, Figure S3G). Notably, all three are known regulators of cell aggregation and proliferation and were found embedded within *MYC*-carrying ecDNA region (Figure S4) 63, 64. To directly compare amplicon structure within 3D culture, we reconstructed the *MYC* region topology in representative replicates (DM_3D-2 and HSR_3D-2) using AmpliconArchitect. In DM_3D-2, the amplified region was maintained predominantly as a highly amplified structure, inferred to be composed of high copy-number circular elements. (Figure S4A). In contrast, HSR_3D-2 amplicon showed substantially lower overall coverage scale and the analysis provided little supported evidence of a closed circular organization (Figure S4B). Detailed inspection of the reconstructed topology then confirmed that the aggregation-associated lncRNAs (*PCAT1, PRNCR1*, and *CASC19)*, whose copy-number gains strongly correlated with cell aggregation were physically embedded within these inferred high CN circular ecDNA structures (Figure S4C). The differential organization and high amplification of *MYC*-carrying ecDNA in the 3D collagen model strongly correlate with the enhanced aggregation phenotype observed in DM_3D.

Overall, the results demonstrate that *MYC*-carrying ecDNA retains its core structure and gene content within the 3D collagen-based hydrogel culture system. Moreover, the strong correlation between cell aggregate formation and oncogene copy number further suggests that this platform can be used to study how ecDNA-driven gene amplification contributes to 3D-specific tumor behaviors. Therefore, this collagen-based 3D culture system can provide a robust platform for investigating ecDNA-associated cellular dynamics in physiologically relevant conditions.

### Cell aggregation patterns and ecDNA-related oncogene expression in a 3D collagen model

Based on our prior confirmation that ecDNA structures are preserved within the 3D collagen cell-culture system, we next investigated whether this system could serve as a 3D culture platform to examine various cellular behaviors of COLO320-DM and COLO320-HSR cells, which harbor distinct ecDNA copy numbers. Specifically, we focused on cell aggregation patterns and oncogene expression within the 3D hydrogel environment.

Cell proliferation, as determined by CCK-8 assay, exhibited a gradual increase in both groups, with DM_3D showing significantly higher metabolic activity than HSR_3D (Figure 3D). However, both COLO320 cell lines encapsulated in the collagen hydrogel consistently maintained their aggregated morphology up to day 28, as shown in Figure S5A. The diameter of cell aggregates and the total area occupied by cell aggregates of Figure 3A significantly increased over time, and a broader size distribution was observed on day 28 compared to day 7 in both groups (Figure S5B-C). The overall aggregate diameter distributions of HSR_3D and DM_3D did not show a significant overall difference (Figure S5B). Because distributional differences were diluted by the large number of small aggregates, we further analyzed the percentage and area of aggregates with diameters larger than 300 μm per unit area (Figure 3E-F). This cutoff was selected because spheroids exceeding this size are reported to develop hypoxic and necrotic cores characteristic of solid tumors 65. DM_3D cultures contained a higher proportion of large aggregates (300 µm), and these aggregates occupied a significantly greater area per unit area compared with HSR_3D cultures. These findings demonstrate that COLO320DM cells preferentially form large, tumor-like aggregates in the 3D collagen culture model. These results indicate that COLO320-DM cells display relatively more tumor-like aggregates and enhanced proliferative activity and aggregation behavior within the 3D collagen culture model. Furthermore, as previously mentioned, the formation of cancer cell aggregates can promote peritumoral vascularization and facilitate metastasis. Therefore, it can indicate that tumors formed by COLO320-DM cells may develop into a more aggressive cancer phenotype.

To evaluate the RNA expression dynamics of oncogenes in this 3D collagen model, we selected the top four oncogenes (*PVT1, MYC, POU5F1B*, and *FAM84B*) with the highest copy numbers present on the ecDNA as shown in Figure 3C. Overall, gene expression levels were consistently higher in COLO320-DM cells than in COLO320-HSR, reflecting the influence of ecDNA amplification. Both *PVT1* and *MYC* showed elevated expression in 3D compared to 2D culture conditions up to day 14. Interestingly, after day 14, *PVT1* expression declined from its peak level as well as *POU5F1B* and *FAM84B* (Figure S5D-H), whereas *MYC* expression continued to increase (Figure 2K). This temporal divergence may be attributed to increasing cell density and aggregation formation in the 3D environment, potentially resulting in hypoxic core regions that influence gene regulation. Similar expression patterns have been previously reported in long-term 3D cultures 66, 67. Additionally, to examine early adaptive events of cancer cells to different cultivation environments, ecDNA-related oncogene expression changes were analyzed within 48 h after encapsulation in collagen hydrogel (3D culture) or seeding on a culture dish (2D culture) (Figure S5I-J). These results suggest that early-stage adaptation is accompanied by rapid and complex transcriptional dynamics in tumor cells.

The sustained upregulation of *MYC* despite the downregulation of *PVT1* may be related to promoter competition at the shared 8q24.21 locus. *PVT1* is known to stabilize MYC by preventing its degradation. As a result, the two genes can enhance each other's expression through a positive feedback loop 68, 69. However, their relationship is not purely cooperative, as they also compete for enhancer elements. The *PVT1* promoter may compete with the *MYC* promoter for shared enhancer interactions in the early stages of tumorigenesis, thereby limiting *MYC* expression (Figure 3G). As the cancer progresses, the *PVT1* promoter can become inactivated due to epigenetic silencing or structural alterations. When this occurs, enhancers are more likely to engage with the *MYC* promoter instead, leading to increased *MYC* expression and accelerated tumor growth 70, 71. Previous studies suggest that the *PVT1* promoter can act as a boundary element, restricting enhancer access to the *MYC* promoter 36, 71. As shown in Figure 3H-I, a decrease in *PVT1* expression may alleviate its insulating effect on the *MYC* locus, thereby facilitating increased *MYC* transcription in 3D culture system. Based on this mechanism and our data, we hypothesized that progressive suppression of PVT1 during culture would lead to elevated MYC expression (Figure 3J-L).

From the results, 3D collagen-based hydrogel systems not only maintain the structural integrity of ecDNA but also promote distinct oncogene expression profiles and aggregation behaviors compared to 2D culture. These differences are likely attributable to the unique physical and spatial constraints inherent to 3D microenvironments. This 3D culture platform offers a foundation for mechanistic investigations into phenomena specific to 3D conditions, such as the relationship between cell aggregate formation and oncogene copy number, as well as the relationship between *PVT1* and *MYC* expression. However, although the results can validate the suitability of collagen-based 3D models for studying ecDNA-driven oncogenic processes, further development of 3D cell-culture platforms should be required because this bulk collagen model often limit nutrient and oxygen diffusion, restrict drug penetration, and fail to support dynamic cell-cell and cell-ECM interactions, thereby insufficiently recapitulating the complex tumor microenvironment.

### Advanced 3D porous collagen cell-culture model with tailorable stiffness

**Figure 4:**
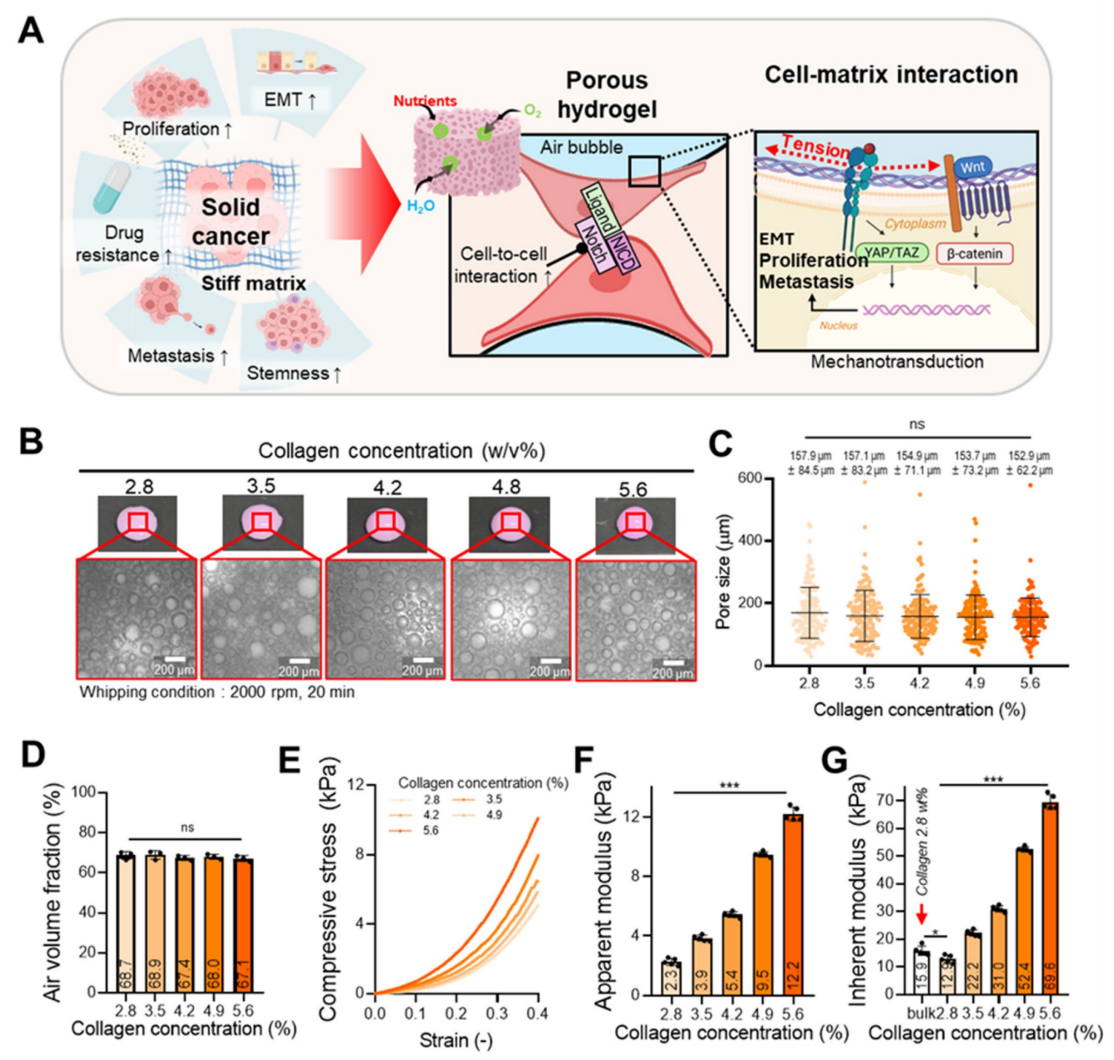
** Characterization of porous collagen foam for various collagen concentrations.** (**A**) Schematic illustration showing the interaction between colorectal cancer cells an ECM, along with the cellular signaling pathways activated within the porous foam. (**B**) Optical images of collagen foams fabricated with different collagen concentrations (2.8-5.6 w/v%). (**C**) Quantification of pore sizes and (**D**) air volume fraction (n = 3) across the different collagen concentrations. (**E**) Compressive stress-strain curves of the collagen foams. (**F**) Apparent compressive modulus and (**G**) inherent modulus estimated using the Gibson-Ashby model (n = 5). All values are represented as mean ± standard deviation.

Building on our previous results showing that 3D collagen culture environments can preserve the structural integrity of ecDNA and induce distinct oncogene expression profiles, we sought to further improve our tumor culture model by modulating matrix stiffness, a critical biophysical feature of the solid tumor microenvironment. In tumor research, the stiffness of the culture matrix has been closely associated with aggressive tumor phenotypes, including enhanced proliferation, cancer stem cell properties, epithelial-mesenchymal transition (EMT), drug resistance, and metastatic potential (Figure 4A) 43-45. However, conventional bulk hydrogels with high stiffness often hinder the diffusion of oxygen and nutrients into the inner regions, leading to impaired cell viability and heterogeneous cell activity 52, 53. To address this limitation, porous 3D tumor models have been proposed as a promising alternative. Although numerous studies have attempted to develop such models, few have succeeded in constructing porous structure using cancer cell-laden hydrogels that also provide physiologically relevant stiffness for tumor cells. Therefore, we employed a highly porous collagen foam as an alternative 3D tumor model. Collagen is composed of amino acids with different polarity properties, including relatively hydrophobic residues such as glycine and proline, as well as hydrophilic residues such as hydroxyproline, resulting in heterogeneous polarity on the molecular surface 72, 73. Owing to these characteristics, when collagen hydrogels are mixed with air during the whipping process, collagen molecules can adsorb at the air-liquid interface, thereby contributing to bubble formation and stabilization of the foam structure. The interconnected porous architecture of porous structures can support improved diffusion of nutrients and gases and facilitates spatial confinement, which enhances cell-cell interactions and promotes mechanotransduction 55. These unique features can allow for sustained cellular activity and effectively modulate oncogene expression.

A porous collagen cell culture matrix was fabricated using varying collagen concentrations (2.8, 3.5, 4.2, 4.8, and 5.6 w/v%) to produce structures with distinct stiffness levels. Optical imaging confirmed that all groups exhibited similar porous morphologies (Figure 4B), with no significant differences in pore size (Figure 4C) or air volume fraction (Figure 4D). To preserve the foam architecture during model fabrication, each bioink was supplemented with 0.3 mM genipin as a pre-crosslinker. In addition, the collagen hydrogel (3.5 w/v%) underwent temperature-induced fibrillogenesis at 37 °C (Figure S6A) 71. Based on this property, the constructs were cast using the mold shown in Figure S6B and subsequently incubated at 37 °C to promote collagen fibrillization, while genipin pre-crosslinking stabilized the foam structure during the crosslinking process. However, as demonstrated in Figure S6B(i-iii), constructs prepared with only the pre-crosslinker, without subsequent crosslinking, failed to maintain their structural integrity (i). In contrast, constructs fabricated without the pre-crosslinker exhibited pore collapse during crosslinking, resulting in pronounced structural deformation (ii). Therefore, structural stabilization of the fabricated foam constructs was ultimately achieved through final crosslinking by immersion in 1 mM genipin (iii). The enhanced structural stability after crosslinking was confirmed by frequency sweep analysis (Figure S6C). After crosslinking, both the storage modulus (G′) and loss modulus (G″) increased markedly, indicating that genipin-mediated crosslinking resulted in increased stiffness of the foam collagen hydrogel.

Compressive stress-strain testing revealed a stiffness-dependent increase in apparent modulus (Figure 4E-F). To better capture the mechanical cues experienced by cells within the porous scaffold, we additionally calculated the inherent modulus using the Gibson-Ashby model 56, which considers the influence of the collagen network's structural confinement (Figure 4G) 13. Here, the inherent modulus refers to the stiffness of the collagen hydrogel matrix present between bubbles.

**Figure 5:**
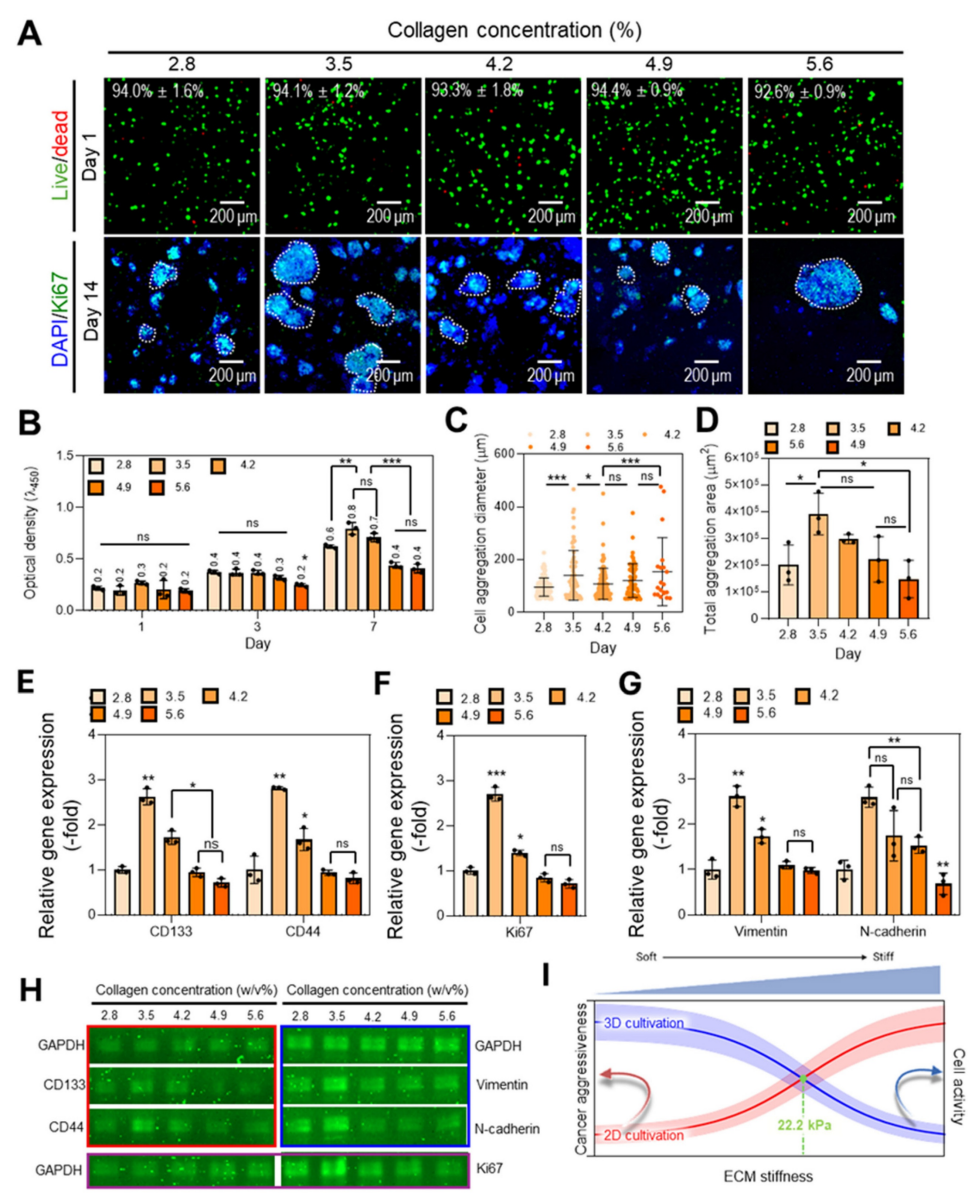
** Effects of collagen foam stiffness on colorectal cancer cell activity.** (**A**) Live (green)/dead (red) fluorescence images and cell viability (n = 3) of cells cultured in collagen hydrogels with varying concentrations. Immunofluorescence images of DAPI (blue)/Ki67 (green) staining at day 14. (**B**) Cell proliferation measured by CCK-8 assay for different collagen concentrations (n = 3). (**C**) Measurement of cancer cell aggregation size (μm) in each condition. (**D**) Total aggregation area per unit area at day 14 (n = 3). Relative gene expression of (**E**) cancer stem cell markers (*CD133, CD44*), (**F**) a proliferation marker (*Ki67*), and (**G**) epithelial-mesenchymal transition (EMT) markers (*Vimentin, N-cadherin*). (**H**) Gel electrophoresis showing expression levels about cancer stem cell markers, proliferation marker, and EMT markers at days 14. (**I**) Graphical representation of ECM stiffness-dependent cellular responses. All values are represented as mean ± standard deviation.

To investigate the biological impact of stiffness variation, we cultured COLO320-DM within the porous collagen matrices and evaluated their behavior over time. Live (green)/dead (red) staining on day 1 showed high viability (> 92%) in all groups. On day 14, DAPI/Ki67 staining confirmed active proliferation and the formation of cell aggregates across all conditions (Figure 5A). Metabolic activity, as assessed by the CCK-8 assay, was highest in the 3.5 w/v% group (Figure 5B), suggesting that this stiffness level provides appropriate conditions for cell growth. Although cells in the 5.6 w/v% foam formed larger aggregates (Figure 5C), the number of aggregates was significantly lower, indicating that excessive confining stress can hinder aggregate formation by limiting proliferation and inducing apoptosis 74. Caspase-3 activity was higher in the 5.6 w/v% group compared with the 3.5 w/v% group, suggesting increased apoptotic activity in the 5.6 w/v% condition (Figure S6D). In addition, the total aggregation area (DAPI/Ki67) shown in Figure 5A was the largest in the 3.5 w/v% group (Figure 5D), further supporting the enhanced aggregation behavior under this intermediate stiffness condition (3.5 w/v% porous collagen).

We next examined stiffness-dependent gene expression associated with tumor progression (Figure 5E-H). The cancer stem cell markers CD133 and CD44 showed their highest expression levels in the 3.5 w/v% group (Figure 5E), consistent with enhanced stemness at this stiffness. Ki67, a well-established marker of proliferation, also peaked at this concentration (Figure 5F), corroborating both immunofluorescence and metabolic activity data. Additionally, expression of the mesenchymal markers Vimentin and N-cadherin was also maximized at 3.5 w/v% (Figure 5G), suggesting that epithelial-mesenchymal transition (EMT)-related processes were promoted under these mechanical conditions. These RT-PCR results were further validated by electrophoresis images (Figure 5H).

Although many studies have reported that increased ECM stiffness enhances cancer aggressiveness, the results are primarily based on 2D culture models 75. In contrast, within 3D environments, cells are subjected to mechanical compression from all directions by the surrounding ECM. This spatial confinement may increase cellular stress and damage, leading to reduced cell activity 76. Therefore, when culturing cancer cells in 3D environments, it is important to identify an optimal stiffness point that balances stiffness-induced cancer aggressiveness with stiffness-mediated suppression of cell activity (Figure 5I). However, this optimal stiffness point may vary depending on the type and structural characteristics of the hydrogel and the specific cell type used. In our system, we observed that at stiffness above 22.2 kPa, both cell proliferation and cell aggregate formation decreased. Below 22.2 kPa, cell proliferation did not differ significantly, while tumor cell aggressiveness was relatively lower. Therefore, we considered 22.2 kPa to be a cross-point between increasing cancer aggressiveness and decreasing cell activity as matrix stiffness increased.

Taken together, the results demonstrate that the porous collagen matrix with intermediate stiffness (3.5 w/v%) provides a mechanically favorable environment for supporting colorectal cancer cell proliferation, aggregation, stem-like behavior, and EMT-related gene expression. This platform can offer a physiologically relevant system for studying the interplay between matrix mechanics and tumor progression in 3D culture models.

### Comparison of COLO320 cell-laden porous and bulk 3D collagen models

**Figure 6:**
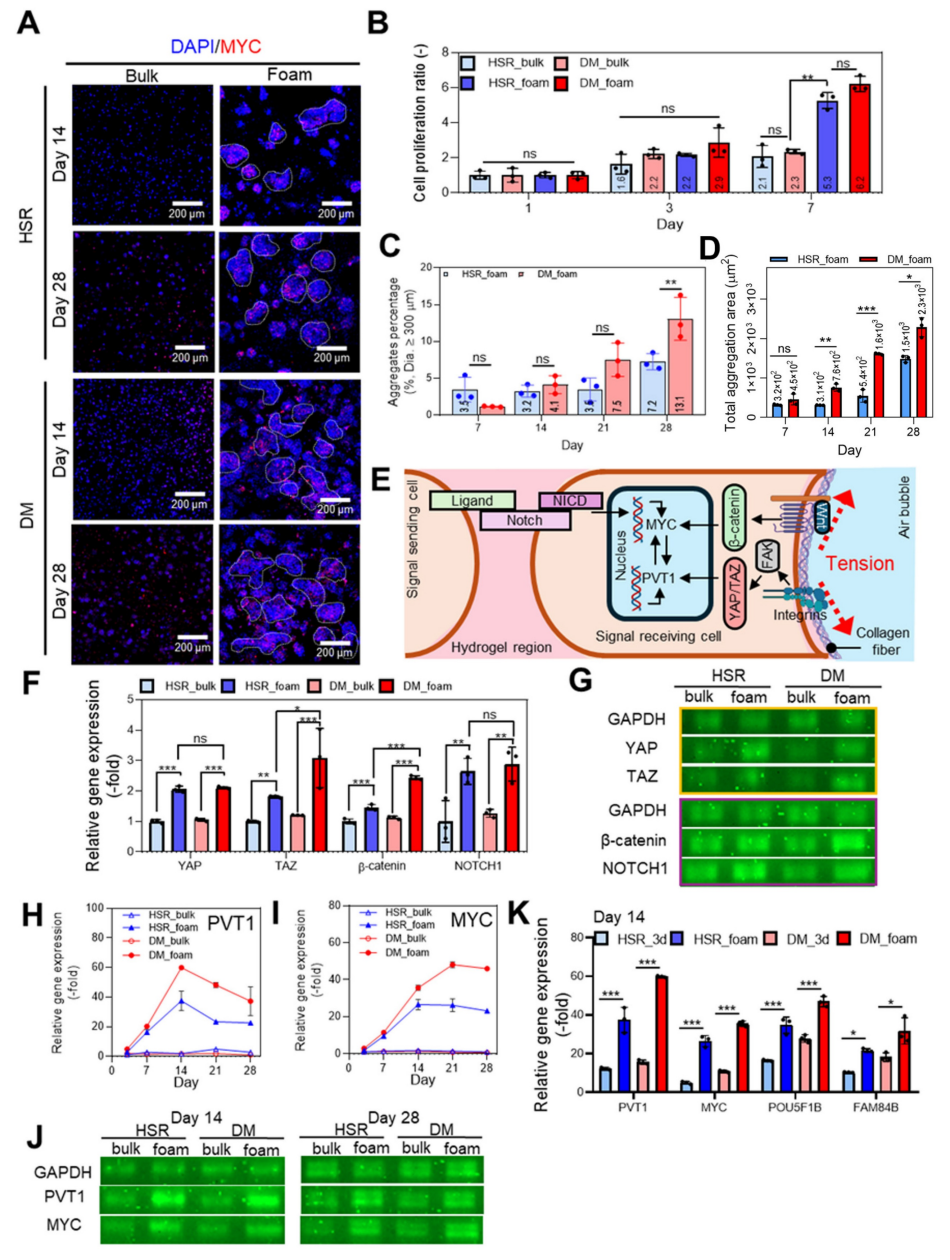
***In vitro* cellular analysis of COLO320 laden in collagen foam bioinks.** (**A**) Immunofluorescence images of DAPI (blue)/MYC (red) staining of COLO320 cells cultured in collagen bulk and foam bioinks at days 14 and 28. (**B**) Quantification of cell proliferation using CCK-8 assay at days 1, 3, and 7 (n = 3). (**C**) Aggregate percentage based on the number of aggregates with diameters larger than 300 μm in 3D cultures at days 7, 14, 21, and 28. (**D**) Total aggregation area per unit area for aggregates larger than 300 μm in diameter at days 7, 14, 21, and 28 (n = 3). (**E**) Schematic illustration of notch signaling and mechanotransduction pathway in colorectal cancer cell within foam tumor model. (**F**) Relative gene expression (n = 3) and (**G**) gel electrophoresis of notch signaling (Notch1) and mechanotransduction (*YAP, TAZ*, and *β-catenin*) related genes at day 7. Relative RNA expression levels of (**H**) *PVT1* and (**I**) *MYC* over time (days 7, 14, 21, and 28). (**J**) Electrophoresis analysis of gene expressions for *PVT1* and *MYC* at days 14 and 28. (**K**) Comparison of RNA expression levels of *PVT1, MYC*, *POU5F1B*, and *FAM84B* at day 14 between the porous collagen foam model and the minimum collagen concentration-based bulk model for structural maintenance. All values are represented as mean ± standard deviation.

To investigate the influence of matrix architecture on oncogene activation under a consistent stiffness condition, COLO320-HSR and COLO320-DM cells were laden in either porous collagen matrix or bulk-type collagen matrix, both fabricated at the properly selected collagen concentration of 3.5 w/v%. The inherent modulus of the bulk and foam constructs was 26.4 ± 1.58 kPa and 21.7 ± 2.68 kPa, respectively (Figure S6E-F). Immunofluorescence staining of DAPI/MYC (red) (Figure 6A) revealed substantially enhanced nuclear expression and clustered localization in foam matrix compared to bulk hydrogels, particularly in COLO320-DM cells. In contrast, minimal cell aggregation was observed in the bulk hydrogels, likely due to limited oxygen and nutrient diffusion associated with the dense matrix architecture.

Consistent with these observations, metabolic activity measured by the CCK-8 assay was significantly higher in foam constructs than in bulk counterparts (Figure 6B). Figure S7A shows that both COLO320 cell lines remained proliferative within the collagen hydrogel up to day 28. In agreement with prior results (Figure S5B-C), the diameter of cell aggregates and quantification of the aggregate-covered area per imaging field were slightly larger in the DM_foam group (Figure S7D-E). DM_foam exhibited a higher proportion of aggregates larger than 300 μm, and the aggregate-covered area per unit area was significantly higher than that of the HSR_foam group (Figure 6C-D), suggesting that the porous tumor model effectively distinguishes phenotypic differences between ecDNA-high and ecDNA-low cancer cell populations.

The architecture of the porous collagen matrix may enhance cell stretching and cell-cell interactions, thereby facilitating oncogene expression through mechanotransduction induced by the curvature of the porous surface, as shown schematically in Figure 6E 55, 77. Such mechanical cues are known to activate several mechanotransduction pathways including *YAP/TAZ* and *Wnt/β-catenin* signaling (Figure 6F-G), both of which promote transcription of *PVT1* and *MYC* 78-80. These two genes also reinforce each other *via* a positive feedback mechanism 68, 69. To more evaluate the contribution of cell-collagen interactions to mechanotransduction, α2β1 integrin was inhibited using BTT-3033 to suppress cell-collagen binding 81. As a result, the expression of *YAP/TAZ* was reduced, accompanied by decreased expression of *MYC* and *PVT1* (Figure S7B-C). These results indicate that mechanotransduction mediated by cell-collagen interactions contribute to the regulation of oncogene expression. In addition, Notch signaling, which requires direct contact between ligand-presenting and receptor-expressing cells, may further contribute to *MYC* transcription. Indeed, the expression of key genes associated with these signaling pathways was upregulated in foam cultures compared to bulk scaffolds (Figure 6H-J). As previously observed, *PVT1, POU5F1B*, and *FAM84B* expressions peaked at day 14 and declined thereafter, whereas *MYC* expression remained elevated over time, reaching a plateau (Figure 6H-J and Figure S7F-H). In addition, *MYC* and *PVT1* expressions were examined within 48 h after encapsulation in bulk and foam collagen hydrogels (Figure S7I-J). Consistent with the earlier observations (Figure S5I-J), these results suggest that rapid mechano-genomic and transcriptomic reprogramming may occur during this early period.

The difference with bulk and foam models may be attributed to restricted cellular activity imposed by the bulk structure. To further test the impact of architecture, we compared fold-changes from day 1 to day 14 in conventional 2% bulk collagen; expression of *PVT1, MYC, POU5F1B,* and *FAM84B* was markedly higher in foam scaffolds (Figure 6K). Expression levels of both *PVT1* and *MYC*, as well as *POU5F1B* and *FAM84B*, were markedly higher in foam scaffolds, confirming that matrix porosity, rather than stiffness alone, plays a critical role in activating ecDNA-associated oncogenes.

## Discussion

In this study, we developed a tailorable 3D porous collagen hydrogel model that closely mimics the tumor microenvironment and enables physiologically relevant cell-cell and cell-ECM interactions. Traditional 2D tumor cell culture systems have been widely used for ecDNA studies and can preserve amplified structures 82-84, but they fail to recapitulate the spatial and mechanical complexity of the 3D TME. This limitation has hindered mechanistic investigations of how ecDNA-driven oncogene dynamics evolve under physiologically relevant 3D conditions.

Using our collagen-based 3D cell-culture system, we demonstrated that ecDNA structures carrying important oncogenes, including *MYC* and *PVT1*, are stably maintained in both DM and HSR types of COLO320 cells. Notably, DM cells exhibited more dynamic structural changes under 3D conditions, whereas HSR cells largely preserved their ecDNA structures. The results indicate that ecDNA behavior can be shaped by both intrinsic cellular features and the surrounding culture environment.

At the transcriptional level, we observed a sustained upregulation of *MYC* expression in the 3D collagen culture system, consistent with ecDNA maintenance and enhancer reorganization mechanisms described in previous studies 36, 71. Early during tumorigenesis, the *PVT1* promoter may compete with the *MYC* promoter for shared enhancer interactions, limiting *MYC* expression. As cancer progresses, epigenetic silencing or structural alterations can inactivate the *PVT1* promoter, enabling enhancers to preferentially engage MYC, thereby driving oncogene activation and tumor progression 70, 71. Although this mechanism has been proposed, further mechanistic validation is needed to elucidate its role under 3D conditions. In addition, the early adaptation phase also appears to involve complex and rapid mechano-genomic and transcriptomic reprogramming. In particular, *MYC* has been reported to exhibit highly dynamic expression changes during the early stage (0-72 h) at the single-cell level 85, suggesting that further detailed investigation of early adaptive dynamics is needed.

To further optimize the model, we engineered a porous collagen matrix with tailorable stiffness, reflecting the progressive stiffening of the tumor microenvironment during cancer development. Increased stiffness can promote tumor growth while generating compressive stress that activates mechanotransduction pathways through cell-ECM receptors such as integrins 86, 87. We found that the porous tumor model supported higher cellular proliferation, aggregation, and metabolic activity than bulk model, and enhanced the expression of ecDNA-associated oncogenes (e.g., *MYC, PVT1, POU5F1B, FAM84B*), particularly in ecDNA-high cells. These indicate that matrix porosity, independent of stiffness, can play a key role in regulating cancer cell behavior and provide a more responsive platform for evaluating ecDNA-driven tumor heterogeneity.

The interconnected porous network of the matrix also facilitates nutrient diffusion, endothelial capillary-like structure formation, and immune cell infiltration and migration. Such features are essential for establishing physiologically relevant tumor models that replicate angiogenesis, immune interactions, and spatial tissue organization, thereby enabling advanced studies on tumor progression and therapeutic responses.

Despite these advances, some limitations remain. First, the use of only two colorectal cancer cell lines may limit the generalizability of the findings, although these models were chosen for their well-defined ecDNA architectures to enable robust platform validation. Second, AmpliconArchitect, which relies on short-read sequencing, cannot always distinguish chromosomal from extrachromosomal amplifications; long-read sequencing will be crucial to resolve ecDNA structures and their circular topology more accurately 88. Additionally, the absence of RNA-seq data limited our ability to directly correlate copy-number variations with transcriptional outputs, particularly for aggregation-related lncRNAs such as *PCAT1, PRNCR1*, and *CASC19*. Integrating transcriptomic and structural analyses in future work will provide deeper insights into ecDNA-driven transcriptional programs under 3D conditions.

Overall, our 3D porous collagen model provides a reproducible and physiologically relevant platform for investigating ecDNA biology in colorectal cancer. By combining tailorable mechanical properties with enhanced cell-matrix interactions, this model captures key features of the TME and enables the long-term study of ecDNA dynamics and oncogene regulation. Its compatibility with advanced analytical and microfluidic systems expands its utility, offering broad potential for future mechanistic studies, therapeutic screening, and tumor evolution research.

## Conclusion

We developed a tailorable 3D porous collagen hydrogel that more accurately mimics the tumor microenvironment and enables stable long-term maintenance of ecDNA in colorectal cancer models. This platform enhanced cellular proliferation, aggregation, and ecDNA-associated oncogene expression compared to bulk hydrogels, revealing distinct ecDNA dynamics between DM and HSR cells. Although further multi-omics analyses are needed, this porous 3D system provides a robust and physiologically relevant foundation for studying ecDNA behavior and tumor evolution, with strong potential for future mechanistic and therapeutic applications.

## Supplementary Material

Supplementary figures and tables.

## Data Availability

The data that support the findings of this study are available from the corresponding author upon reasonable request.

## Abbreviations

TME: tumor microenvironment
ECM: extracellular matrix
ecDNA: extrachromosomal DNA
CRC: colorectal cancer
FBS: fetal bovine serum
PS: penicillin-streptomycin
3DW: tiple-distilled water
cDNA: complementary DNA
WGS: whole-genome sequencing
BQSR: base quality score recalibration
CoRAL: complete reconstruction of amplifications with long reads
ANOVA: one-way analysis of variance
HSD: honestly significant difference
MYC: MYC proto-oncogene
PVT1: plasmacytoma variant translocation 1
lncRNA: long non-coding RNA
IRF4: interferon regulatory factor 4
CDX2: caudal type homeobox 2
PDX1: pancreatic and duodenal homeobox 1
FLT3: fms-like tyrosine kinase 3
CYLD: cylindromatosis
ChrAmp: chromosomal amplicons
POU5F1B: POU class 5 transcription factor 1B
FAM84B: family with sequence similarity 84, member B
PCAWG: Pan-Cancer Analysis of Whole Genomes
TCGA: The Cancer Genome Atlas
EMT: epithelial-mesenchymal transition
