# Supplementary material for: Tailorable porous collagen hydrogels as a physiologically relevant platform for extrachromosomal DNA-associated colorectal cancer research
Source: Theranostics. 2026 Mar 9;16(10):5240–58. doi: 10.7150/thno.128574 (PMC13080448; doi:10.7150/thno.128574)
Supplement: Supplementary file 1 — Supplementary figures and tables. [file thnov16p5240s1.pdf]

Tailorable porous collagen hydrogels as a physiologically relevant platform for extrachromosomal DNA-associated colorectal cancer research

Seoyul Jo[1,†], Jiwon Shon[2,†], Seohyeon An[1], Yoonjoo Nam[3], Dongwon Choi[4], Seungmi Lee[4], Hoigi Seo[5],

[1]Department of Precision Medicine, Sungkyunkwan University School of Medicine (SKKU-SOM), Suwon 16419, Republic of Korea

[2]Department of Biohealth Regulatory Science, School of Pharmacy, Sungkyunkwan University, Suwon, Korea

[3]Department of Biopharmaceutical Convergence, School of Pharmacy, Sungkyunkwan University, Suwon, Korea

[4]Department of Pharmacy, School of Pharmacy, Sungkyunkwan University, Suwon-si, South Korea

[5]Department of Electrical and Computer Engineering, Seoul National University, Seoul, Korea

[6]INMC & IPAI, Seoul National University, Seoul, Korea

[7]Institute of Quantum Biophysics, Department of Biophysics, Sungkyunkwan University, Suwon, Gyeonggi-do 16419, Republic of Korea

[8]Biomedical Institute for Convergence at SKKU (BICS), Sungkyunkwan University, Suwon 16419, Republic of Korea

[†] These authors contributed equally.

[*]Corresponding authors, Prof. Hoon Kim: wisekh@skku.edu; Prof. GeunHyung Kim: gkimbme@skku.edu

Supplementary Tables

Table 1. Various tumor models. Overview of various engineered 3D TME models with a focus on their fabrication purposes

Purpose	Tissue	Cell	Method	Results	Ref.
Drug screening	Colon	Caco-2, patient deriv ed CRC	Cancer cells encapsulation in RGD-functionalized alg inate with nanofibrillar cellulose → 3D bioprinting → crosslinking with $CaCl_2$ in PBS	-Better mimicking of *in vivo* cellular behavior than 2D culture<br>-Validation as a platform for testing chemotherapeutic responses	[15]
		HCT116, HUVEC, HELF	3D printing of mesh-structured PCL scaffolds → 0.1 w t/v% collagen coating → seeding with cancer, fibrobla st, and endothelial cells	-Better mimicking of *in vivo* cellular behavior than 2D culture<br>-Validation as a platform for testing chemotherapeutic responses<br>-Co-culturing with stromal cells (endothelial cells and fibroblast s)	[8]
		Patient derived CRC and CAF	CRC organoids encapsulation in thiol-modified HA/g elatin hydrogels → seeding with CAFs on top of the C RC-laden hydrogels.	-Validation as a platform for testing chemotherapeutic responses<br>-Co-culturing with stromal cell (fibroblasts)	[16]
	Pancreas	BxPC-3, Panc-1, pati ent-derived pancreati c cancer cells and C AF	Mixed cells (cancer cells:CAFs = 1:1) encapsulation i n oligomeric collagen → embedding in a collagen-bas ed tissue matrix within 96-well plates.	-Validation as a platform for testing chemotherapeutic responses<br>-Co-culturing with stromal cell (fibroblasts)<br>-Simple, rapid, and reproducible model creation with user custo mization	[9]
Comparative ana lysis	Colon	HT-29, SW480, HC T116	Cancer cells encapsulation in PEGDA/fibrinogen hydr ogels → crosslinking under visible light exposure.	-Better mimicking of *in vivo* cellular behavior than 2D culture<br>-High potential for further cell-line dependent investigation	[10]
Mechanism inve stigation	Colon	Caco-2	Cancer cells encapsulation in a bioinspired GHP_{4a} hyd rogel → photo-crosslinking by LED light	-Better mimicking of *in vivo* cellular behavior than 2D culture<br>-Investigation of the mechanism promoting anoikis resistance th rough activation of the FAK/PI3K–Akt signaling pathway	[11]
		Patient derived CRC	CRCs encapsulation in a Me-HA hydrogel → UV cros slinking	-Investigation of TRPV4–PI3K/Akt–HSF1–Hsp70 signaling in s tiff hydrogels exhibiting higher tumorigenic and metastatic pote ntial *in vivo*<br>-Implying that Hsp70 may serve as a promising molecular thera peutic target for cancer treatment	[13]
	Pancreas	HPDE6-C7, Panc-1, THP-1	Cancer cells encapsulation in GelMA-HAMA hydroge ls → UV crosslinking → indirect (using transwell cha mber) or direct (mixing with Panc-1 cells) co-culture with macrophages	-Better mimicking of *in vivo* cellular behavior than 2D culture<br>-Investigation of M2-like polarization and enhanced PANC-1 st emness via co-culture with macrophages and activation of the P I3K–AKT–SELE/VCAM1 axis	[12]

				-Co-culturing with immune cell (THP-1)	
	Bone	MG63, 143B, hBM-MSC	Embedding of tumor spheroids in an hBM-MSC-laden PLMA hydrogel within a microfluidic device	-Providing evidence for the role of a dynamic environment as a key regulator of tumor metastatic ability<br>-Co-culturing with stromal cell (hBM-MSC)	[18]
Immunotherapy evaluation	Breast	MCF-7, THP-1	Alginate hydrogel crosslinked by $CaCl_2$ → freeze-drying → seeding with MCF-7 cells and macrophages (THP-1) → adding IL-4 to induce macrophage polarization	-Replacing the conventional bulk culture method for evaluating the therapeutic effects of chemo-immunotherapy<br>-Co-culturing with immune cell (THP-1)	[17]
	Prostate	PC3, HT1080, RM1, UniCAR T cell	Encapsulation of Mono- and co-cultured spheroids (PC3-PSCA or RM1 with HT1080) in PEGDA hydrogel beads using microfluidic UV crosslinking → transfer to 96-well plates for T cell co-culture	-Better mimicking of *in vivo* cellular behavior than 2D culture<br>-Providing reproducible platforms to evaluate immunotherapeutic efficacy, better represent tumor heterogeneity, and support the development of personalized therapies.<br>-Co-culturing with stromal cell (HT1080) and immune cell (UniCAR T cell)	[14]

Abbreviations: GelMA-HAMA-4-arm-PEGDA (GHP4a); human umbilical vein endothelial cells (HUVECs); human embryo lung fibroblasts (HELFs); colorectal cancer cell (CRC); Cancer-associated fibroblasts (CAF); human bone marrow mesenchymal stem cells (hBM-MSC)s; methacryloyl platelet lysate (PLMA); tumor associated macrophages (TAMs); methacrylated hyaluronic acid (MeHA)

Table 2. Primer sequences. Information of the primers used in RT-qPCR.

Gene	Source	Primer sequence	
		Left (5'-3')	Right (3'-5')
GAPDH	Homo sapiens	GTCTCCTCTGACTTCAACAGCG	ACCACCCTGTTGCTGTAGCCAA
PVT1	Homo sapiens	TTACAGGCGTGTGCCACAAAGC	GCCTGTAATCCCAGCACGTTGA
MYC	Homo sapiens	CCTGGTGCTCCATGAGGAGAC	CAGACTCTGACCTTTTGCCAGG
POU5F1B	Homo sapiens	CCTGAAGCAGAAGAGGATCACC	AAGCGGCAGATGGTCTTTTGGC
FAM84B	Homo sapiens	GTGGAATGCTCCGTGTTCTACC	TACTGAGCCTGCGACACGAACT
CD133	Homo sapiens	CACTACCAAGGACAAGGCGTTC	CAACGCCTCTTTGGTCTCCTTG
CD44	Homo sapiens	CCAGAAGGAACAGTGGTTTGGC	ACTGTCCTCTGGGCTTGGTGTT
Ki67	Homo sapiens	GAAAGAGTGGCAACCTGCCTTC	GCACCAAGTTTTACTACATCTGCC
Vimentin	Homo sapiens	AGGCAAAGCAGGAGTCCACTGA	ATCTGGCGTTCCAGGGACTCAT
N-cadherin	Homo sapiens	CCTCCAGAGTTTACTGCCATGAC	GTAGGATCTCCGCCACTGATTC

Supplementary Figures

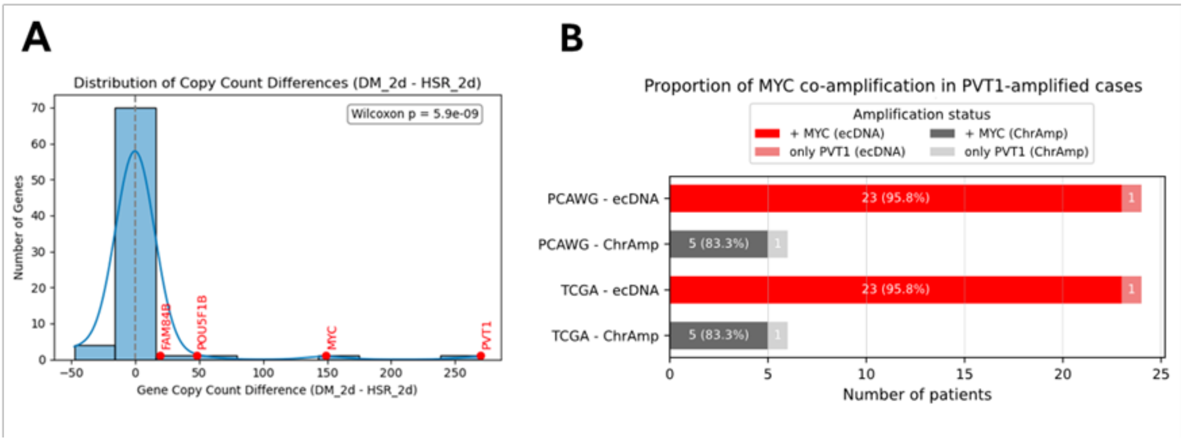

Figure S1. Co-amplification of *PVT1* with *MYC* and gene copy number differences between COLO320 cell lines in 2D cultures. (**A**) Distribution of DNA-level copy-number difference (DM-HSR). *MYC*-associated loci (e.g., *MYC, PVT1, POU5F1B, FAM84B*) fall in the right tails, indicating larger gains in DM relative to HSR. (**B**) Stacked bars summarize *PVT1*-positive tumors in PCAWG and TCGA, stratified by *MYC* status and amplicon type. Categories are: +*MYC* on ecDNA (red) and *MYC* on chromosomal amplicons (dark gray). We also show only *PVT1* on ecDNA (light red) and only *PVT1* on chromosomal amplicons (light gray). Co-amplified *MYC-PVT1* on ecDNA predominated: 76.7% in PCAWG and 73.9% in TCGA.

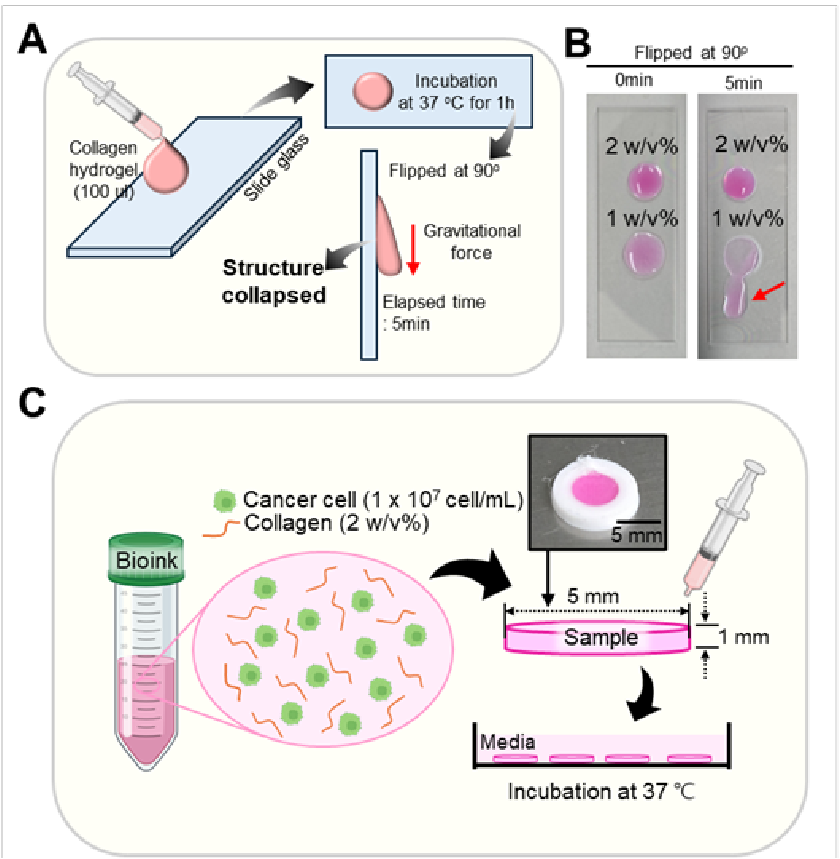

Figure S2. Evaluation of the minimum collagen concentration required for post-gelation structural stability. (A) Schematic representation of the gravitational collapse assay for collagen hydrogels after thermal gelation at 37 °C for 1 h, including vertical inversion and 5 min incubation under gravitational force. **(B)** Optical images of collagen hydrogel drop, tilted at 0 and 90° for 10 min. 1 w/v% collagen hydrogel showing structure collapse (red arrow). **(C)** Schematic of how to fabricate 3D collagen hydrogel sample.

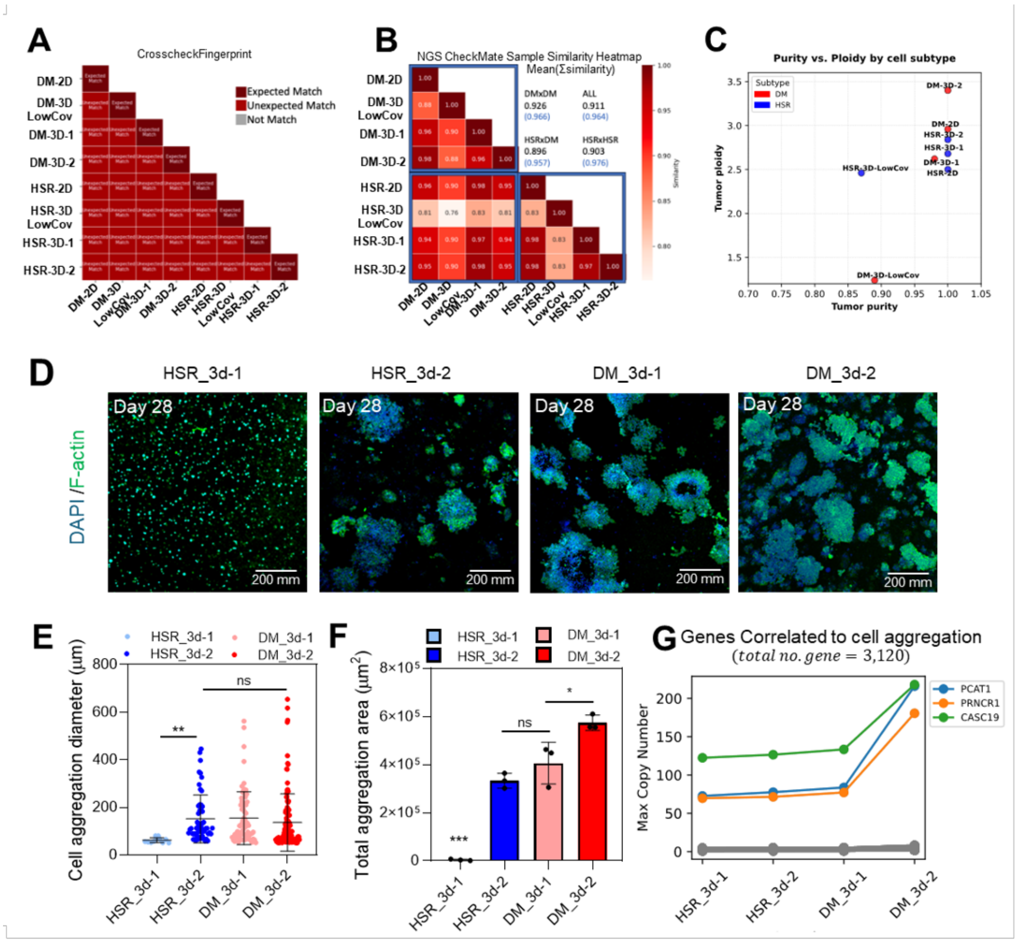

Figure S3. Sample quality assessment across COLO320 WGS dataset and characterization of 3D cultured COLO320 cells. (**A**) Sample-identity validation by Picard CrosscheckFingerprints. All libraries matched their designated COLO320 isogenic cell lines (DM, HSR) across 2D and 3D replicates. No swaps or contamination were detected. "Unexpected matches" refer to pairs that were differently labeled but genetically identical. (**B**) NGSCheckMate sample similarity heatmap (genotype concordance). Within cell line comparison (DM-DM, HSR-HSR) including 2D-3D cross condition pairs, show high concordance, whereas between isogenic cell line comparisons (DM-HSR) are consistently lower. (**C**) Purity and Ploidy scatter for 8 COLO320 WGS libraries estimated by PURPLE and GATK. Points are group by (DM: red; HSR: blue) and culture (2D, 3D). (**D**) DAPI (blue)/F-actin (green) staining images, (**E**) cell aggregation size (um), and (**F**) total aggregation area (μm^2) about four 3D cultured samples (HSR_3d-1, HSR_3d-2, DM_3d-1, and DM_3d-2) at days 28. (**G**) Line plots show the maximum copy number of *PCAT1, PRNCR1*, and *CASC19* across HSR and DM samples. All three lncRNAs exhibit a progressive increase in copy number from HSR to DM samples. The rank order of copy number changes was perfectly correlated among the three genes (Spearman's rho = 1.00), indicating a highly consistent amplification trend across samples. All values are represented as mean ± standard deviation.

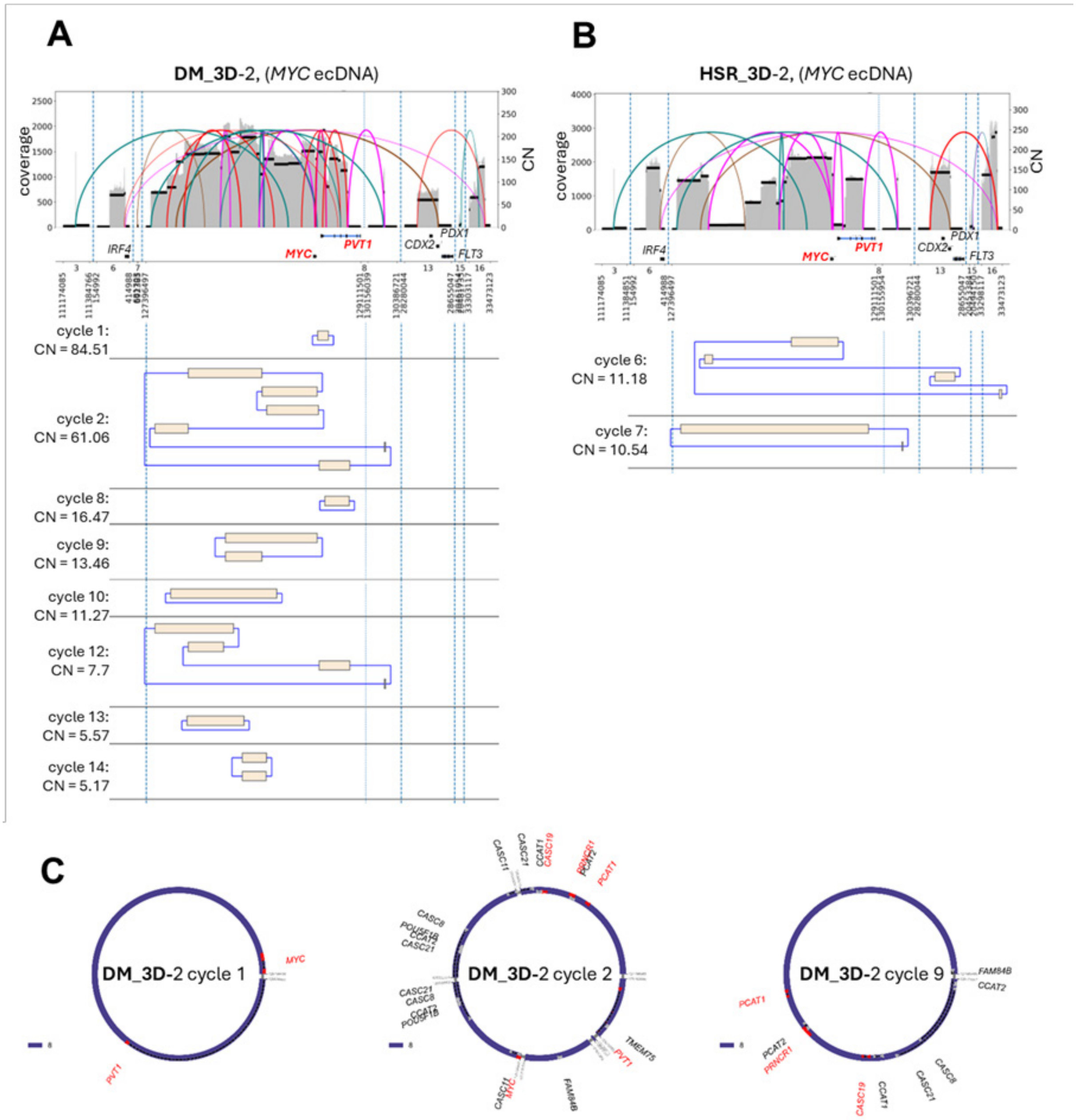

Figure S4. Structural comparison of *MYC* ecDNA topology in 3D cultured COLO320 cells. Amplicon graph visualization by AmpliconArchitect, CoRAL and CycleViz showing structural rearrangements, coverage, copy-number (CN), and breakpoint connections across genomic segments. (**A**) *MYC*-containing amplicon topology in DM_3D-2 . The plot shows coverage on the left y-axis and CN on the right y-axis. The structure exhibits high segment coverage and is composed of multiple high-CN inferred circular elements (including only inferred elements with CN>5), indicating a highly amplified and structurally complex ecDNA population. The term 'inferred cycle' refers to the ecDNA topology reconstructed from short-read sequencing data. (**B**) *MYC*-containing amplicon topology in HSR_3D-2. In contrast to the DM_3D-2, the HSR_3D-2 amplicon showed lower CN inferred cycle elements, suggesting a less organized ecDNA structure. (**C**) CycleViz models of representative inferred

circular elements in DM_3D-2. The models highlight the co-integration of the core *MYC* and *PVT1* locus (red) along with the aggregation-associated lncRNAs (*PCAT1, PRNCR1*, and *CASC19*; also in red). This finding confirms that the increased CN of these regulatory lncRNAs stems from their physical embedding within the *MYC*-ecDNA population in the DM_3D model.

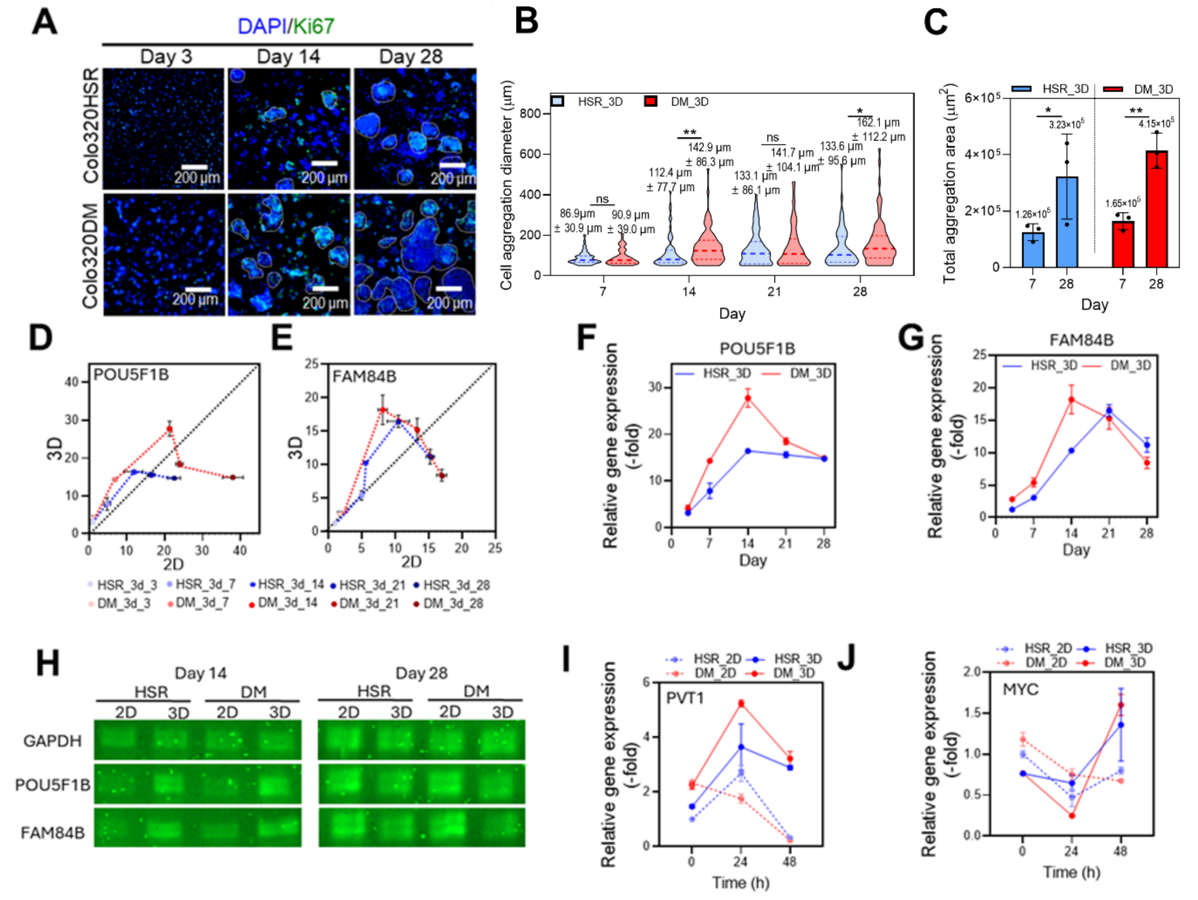

Figure S5. Characterization of cell proliferation and ecDNA associated *POU5F1B* and *FAM84B* in 3D cultured COLO320 cells compared to 2D environments. (**A**) DAPI (blue)/Ki67 (green) staining images at days 3, 14, and 28. (**B**) Distribution of individual aggregation diameter in 3D cultures on days 7, 14, 21, and 28. (**C**) Total aggregation area per unit area at days 7 and 28 (n = 3). Relative mRNA expression of **(D)** *POU5F1B* and **(E)** *FAM84B* in COLO320-DM and HSR cells cultured in 2D and 3D for 28 days (n = 3). Temporal expression patterns of (**F**) *POU5F1B* and (**G**) *FAM84B* in 3D culture at days 3, 7, 14, 21, and 28. (**H**) Gel electrophoresis showing expression levels of *POU5F1B* and *FAM84B* at days 14 and 28 in 2D and 3D culture. Temporal expression patterns of (**I**) *PVT1* and (**J**) *MYC* in 2D and 3D culture at 0, 24, and 48 h. All values are represented as mean ± standard deviation.

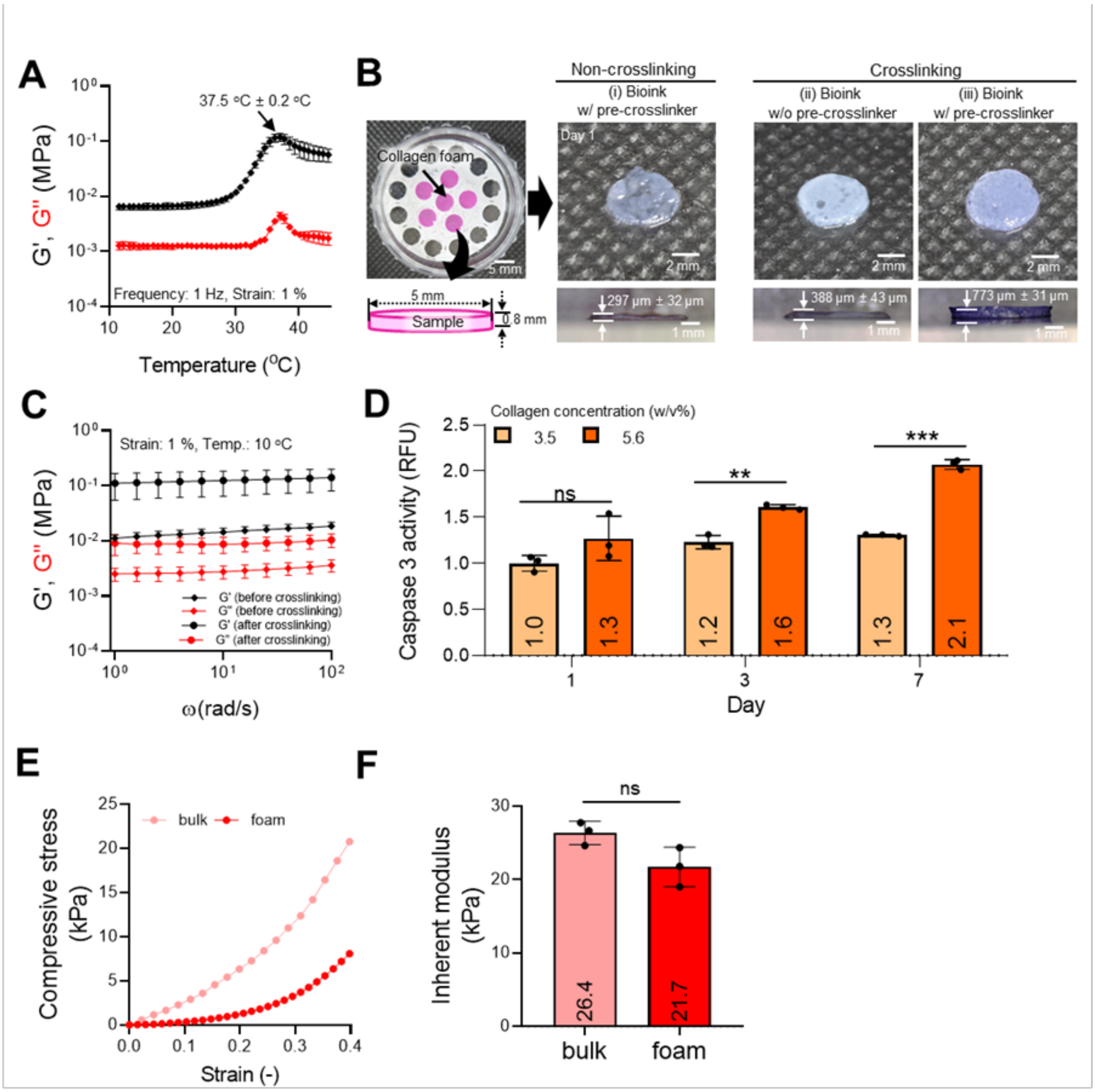

Figure S6. Rheological properties of collagen foam hydrogel after crosslinking and mechanical properties of collagen bulk and foam hydrogels. (**A**) Temperature sweep of bulk collagen hydrogel (3.5 w/v%). (**B**) Optical images of collagen foam structures (height: 0.8 mm, mold diameter: 5 mm) after 24 h under various crosslinking conditions [non-crosslinked collagen constructs: (i) bioink with pre-crosslinker (0.3 mM genipin in collagen foam) and crosslinked collagen constructs: (ii) bioink without pre-crosslinker and (iii) bioink with pre-crosslinker]. (**C**) Frequency sweep (1 - 100 rad/s) showing storage modulus (G') and loss modulus (G") of collagen foam hydrogel before and after crosslinking. (**D**) Caspase-3 activity assay of collagen foams with 3.5 and 5.6 w/v%. (**E**) Compressive stress–strain curves and (**F**) inherent modulus estimated using the Gibson–Ashby model (n = 3) of the collagen bulk and foam hydrogels after crosslinking. All values are represented as mean ± standard deviation.

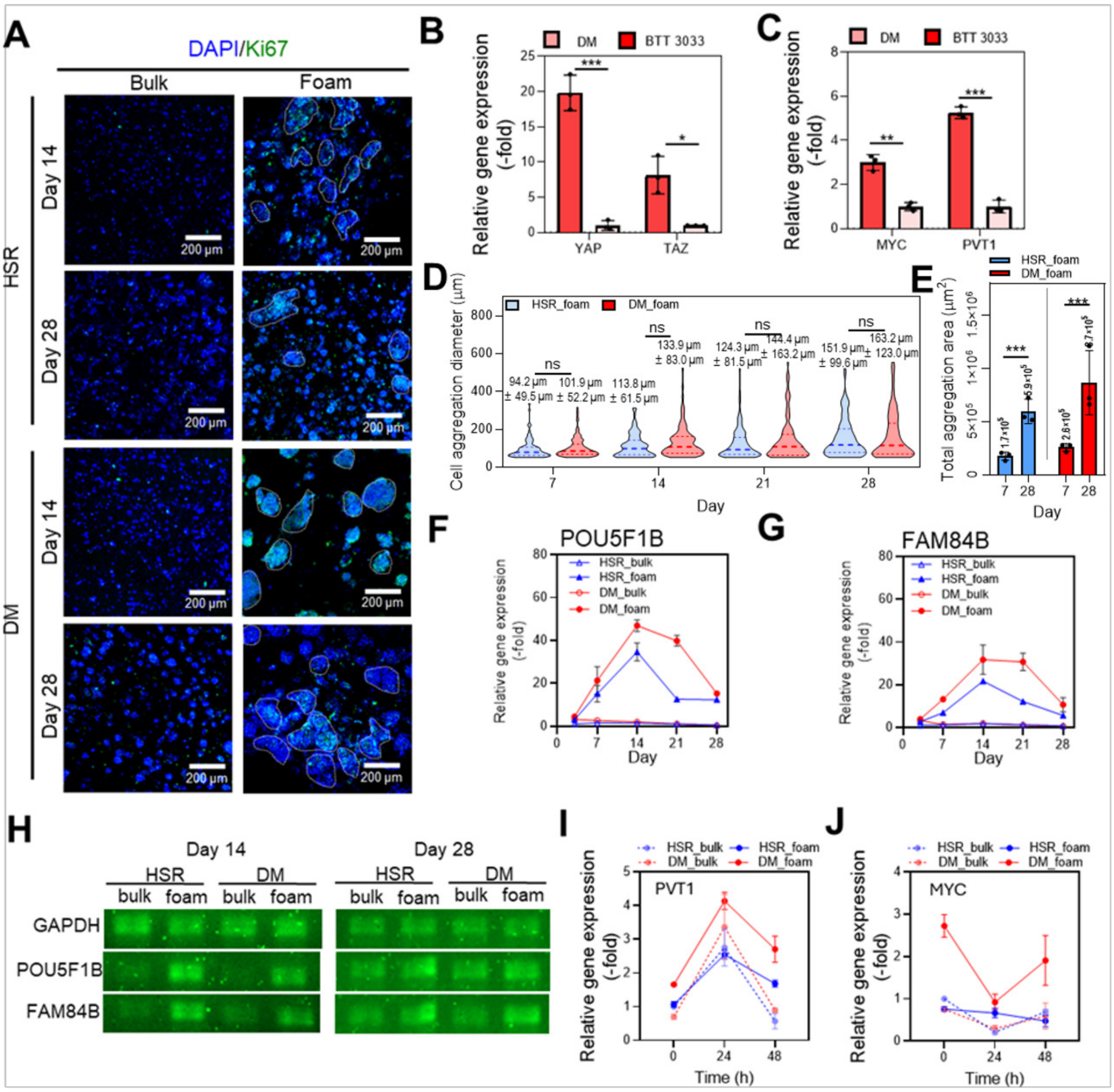

Figure S7. Characterization of cell proliferation and ecDNA associated *POU5F1B* and *FAM84B* in bulk and foam tumor model. (**A**) DAPI (blue)/Ki67 (green) staining images at days 14, and 28. Relative mRNA expression of (**B**) mechanotransduction genes (*YAP* and *TAZ*) and (**C**) ecDNA-related oncogenes (*MYC* and *PVT1*) at day 7. (**D**) Distribution of individual aggregation diameter in 3D cultures on days 7, 14, 21, and 28. (**E**) Total aggregation area per unit area at days 7 and 28 (n = 3). Relative mRNA expression of (**F**) *POU5F1B* and (**G**) *FAM84B* in bulk and foam tumor model at days 3, 7, 14, 21, and 28. (**H**) Gel electrophoresis showing expression levels of *POU5F1B* and *FAM84B* at days 14 and 28. Temporal expression patterns of (**I**) *PVT1* and (**J**) *MYC* in bulk and foam models at 0, 24, and 48 h. All values are represented as mean ± standard deviation.

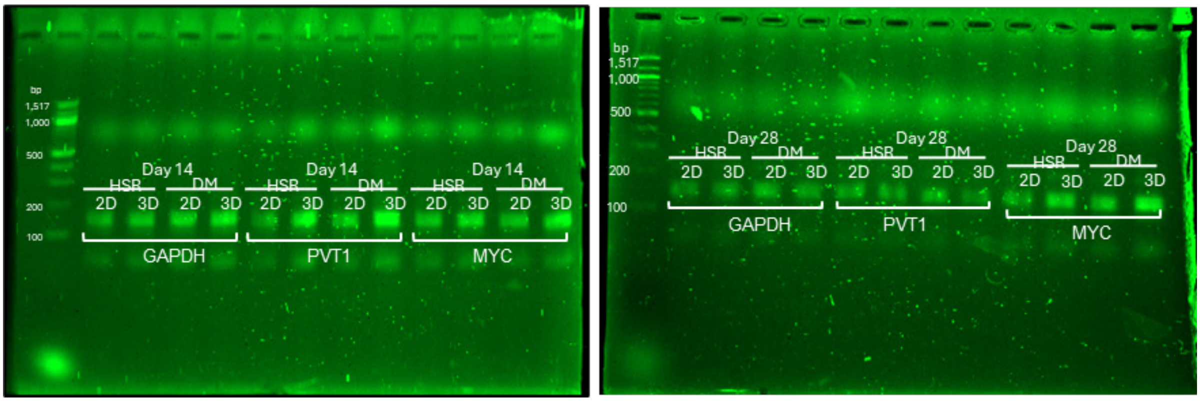

Figure S8. Original cDNA gel electrophoresis data for Figure 3L.

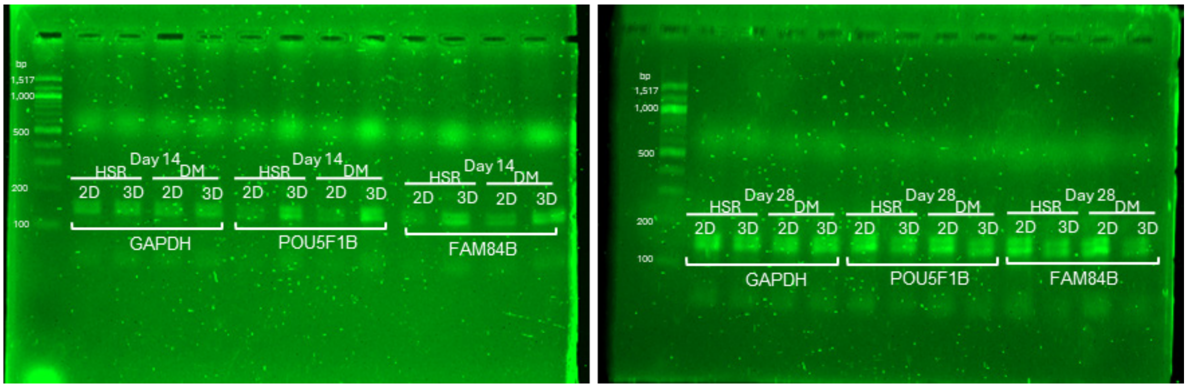

Figure S9. Original cDNA gel electrophoresis data for Figure S5H.

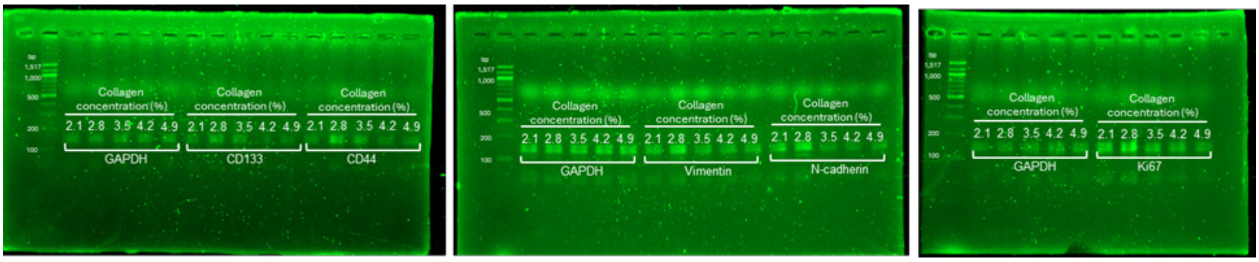

Figure S10. Original cDNA gel electrophoresis data for Figure 5H.

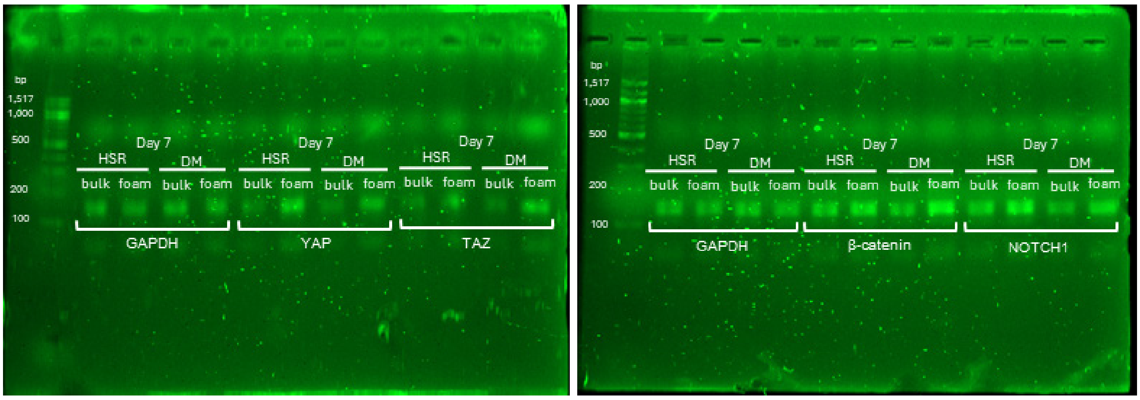

Figure S11. Original cDNA gel electrophoresis data for Figure 6G.

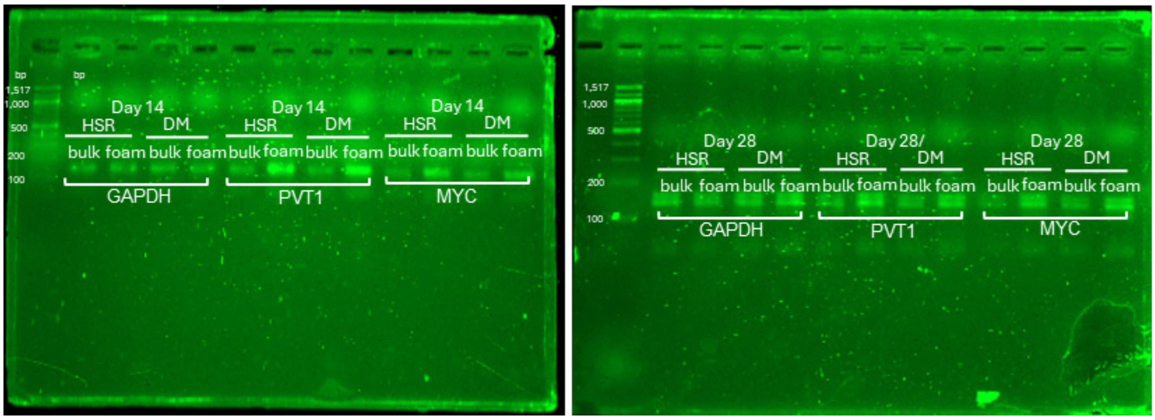

Figure S12. Original cDNA gel electrophoresis data for Figure 6J.

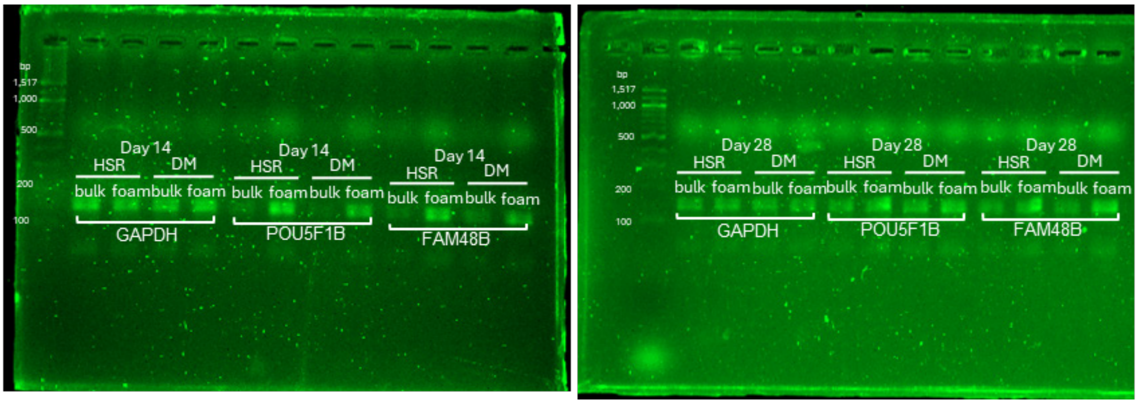

Figure S13. Original cDNA gel electrophoresis data for Figure S7H.
